# *LecRK‐V*, an L‐type lectin receptor kinase in *Haynaldia villosa*, plays positive role in resistance to wheat powdery mildew

**DOI:** 10.1111/pbi.12748

**Published:** 2017-08-01

**Authors:** Zongkuan Wang, Jiangyue Cheng, Anqi Fan, Jia Zhao, Zhongyu Yu, Yingbo Li, Heng Zhang, Jin Xiao, Faheem Muhammad, Haiyan Wang, Aizhong Cao, Liping Xing, Xiue Wang

**Affiliations:** ^1^ State Key Lab of Crop Genetics and Germplasm Enhancement Cytogenetics Institute Nanjing Agricultural University/JCIC‐MCP Nanjing Jiangsu China

**Keywords:** *Haynaldia villosa* L., lectin receptor kinase, *Triticum aestivum* L., powdery mildew resistance, transgenic wheat

## Abstract

Plant sense potential microbial pathogen using pattern recognition receptors (PRRs) to recognize pathogen‐associated molecular patterns (PAMPs). The Lectin receptor‐like kinase genes (*LecRKs*) are involved in various cellular processes mediated by signal transduction pathways. In the present study, an L‐type lectin receptor kinase gene *LecRK‐V* was cloned from *Haynaldia villosa*, a diploid wheat relative which is highly resistant to powdery mildew. The expression of *LecRK‐V* was rapidly up‐regulated by *Bgt* inoculation and chitin treatment. Its transcript level was higher in the leaves than in roots, culms, spikes and callus. Single‐cell transient overexpression of *LecRK‐V* led to decreased haustorium index in wheat variety Yangmai158, which is powdery mildew susceptible. Stable transformation *LecRK‐V* into Yangmai158 significantly enhanced the powdery mildew resistance at both seedling and adult stages. At seedling stage, the transgenic line was highly resistance to 18 of the tested 23 *Bgt* isolates, hypersensitive responses (HR) were observed for 22 *Bgt* isolates, and more ROS at the *Bgt* infection sites was accumulated. These indicated that *LecRK‐V* confers broad‐spectrum resistance to powdery mildew, and ROS and SA pathways contribute to the enhanced powdery mildew resistance in wheat.

## Introduction

Surrounded by pathogens and stimuli, plants recruit a battery of immune mechanisms. The first layer of plant immunity and early response triggered by perception of microbe‐associated molecular patterns (MAMPs) or pathogen‐associated molecular patterns (PAMPs) is called pattern‐triggered immunity (PTI) (Boller and Felix, 2009; Boller and He, 2009; Jones and Dangl, 2006). PTI involves production of reactive oxygen species (ROS), activation of mitogen‐activated protein kinases (MAPKs), callose deposition, changes in gene expression and production of defence compounds (Boller and Felix, 2009; Boudsocq *et al*., 2010; Zhang and Zhou, 2010). To suppress or manipulate PTI, pathogens secret specific effectors in the apoplast or within living plant cells (Jones and Dangl, 2006)**.** The second class of mostly intracellular immune sensors designated resistance (R) proteins, directly or indirectly perceives these effectors and lead to effector‐triggered immunity (ETI). ETI may overlap with PTI, but appears to be accelerated and amplified (Bent and Mackey, 2007; Chisholm *et al*., 2006; Jones and Dangl, 2006; Underwood and Somerville, 2008)**.**

To plants, rapid sensing the potential invaded pathogen is important for activation of defence responses. Plants employ a sophisticate perception and transduction system, which is represented by a large gene family, receptor‐like kinases (RLKs) (Mahajan and Tuteja, 2005). RLKs consist of extracellular domain, transmembrane domain and intracellular kinase domain. They are a vast gene family comprising 610 members in *Arabidopsis*. Lectin receptor kinases (LecRKs), characterized by the presence of an extracellular lectin domain, are one class of RLKs. According to the diversity in lectin motif, LecRKs can be divided into three subclasses: G‐type, C‐type and L‐type (Bouwmeester and Govers, 2009). G‐type LecRKs are famous as S‐domain RLKs and are involved in tremendous amount of mechanisms like self‐incompatibility (Takasaki *et al*., 2000), insect resistance (Liu *et al*., 2014), dark induced leaf senescence (Chen *et al*., 2013). The recently cloned rice brown plant hopper resistance gene *Bph3* contained three G‐type LecRKs encoding genes (*OsLecRK1*‐*OsLecRK3*). *Bph3* has been deployed more than 30 years and still confers broad‐spectrum resistance (Liu *et al*., 2014; Sun *et al*., 2005). Rice Pi‐d2 is a B‐type LecRK and confers resistance against the fungal pathogen *Magnaporthe grisea*, which is the causal agent of rice blast (Chen *et al*., 2006). Classical C‐type lectins contain so‐called carbohydrate recognition domains (CRDs) that bind carbohydrate structure in a calcium (Ca^2+^)‐dependent manner and play a major role in pathogen recognition (Cambi *et al*., 2005). Unlike the omnipresent spread in mammals, C‐type lectin proteins are rare in plants, only one C‐type lectin protein was found in *Arabidopsis*, but its function has not been elucidated.

L‐type LecRKs, which are rich in plant, have an extracellular resemble soluble legume lectin domain. So far, 45 L‐type LecRKs have been identified in *Arabidopsis* (Bouwmeester and Govers, 2009; Herve *et al*., 1996). Most L‐type LecRKs are located on plasma membrane, and they play a putative role in transport extracellular stimuli into intracellular. LecRK‐I.9 was regarded as a potential host target for a RXLR effector and overexpression of *LecRK‐I.9* led to enhanced resistance to *Phytophthora brassicae* (Bouwmeester *et al*., 2011). L‐type lectin receptor kinase‐VI.2 (LecRK‐VI.2) responded to BABA (β‐aminobutyric acid) and positively regulated PTI. The *lecrk‐VI.2‐1* mutant enhanced susceptibility to the hemibiotrophic *Pseudomonas syringae* and the necrotrophic *Pectobacterium carotovorum* bacteria (Singh *et al*., 2012). Overexpression of *AtLPK1* confers disease resistance against the necrotrophic *Botrytis cinerea* (Huang *et al*., 2013). NbLRK1, interacting with INF1 in *Nicotiana benthamiana* via its 31 amino acid region of VIb subdomain of kinase domain, mediated INF1‐induced cell death. A most recent study showed that ectopic expression of Arabidopsis L‐type lectin receptor kinase genes *LecRK‐I.9* and *LecRK‐IX.1* in *Nicotiana benthamiana* confers *Phytophthora* resistance (Wang *et al*., 2016). *Lrk10,* which encoded an L‐type LecRK, was cloned as candidate of the disease resistance locus *Lr10* in wheat (Feuillet *et al*., 1997). These results show that LecRKs play very important roles in plant‐pathogen/insect interactions. However, the function of L‐type LecRK in *Gramineae* is still elusive. Up to now, none of the *LecRKs* gene has been characterized for their function in wheat powdery mildew resistance.

Wheat powdery mildew, caused by *Blumeria graminis* f. sp*. tritici* (*Bgt*), can lead to 13% to 50% yield losses (Griffey *et al*., 1993). *Bgt* is obligate biotrophic, which establishes the infection by getting nutrients from plant via forming haustoria in living epidermal cells. The identification of effective resistance genes is most crucial to wheat breeding for disease resistance. To date, more than 54 powdery mildew resistance loci with 78 genes/alleles, including those introgressed from wild relatives, have been catalogued in wheat (Hao *et al*., 2015; McIntosh *et al*., 2013; Petersen *et al*., 2015). So far, four powdery mildew resistance genes have been cloned: *Pm2* from common wheat (Sánchez‐Martín *et al*., 2016), *Pm3* from common wheat (Bhullar *et al*., 2009; Yahiaoui *et al*., 2004), *Pm8* (Hurni *et al*., 2013, 2014) from rye (*Secale cereal* L.) and a key member of *Pm21*,* Stpk‐V*, from *Haynaldia villosa* (Cao *et al*., 2011).

*Haynaldia villosa*.L(*Dasypyrum villosum*, 2*n* = 14, VV), an annual diploid grass relative of wheat, possesses high level of resistance to several wheat diseases, such as rust, powdery mildew and wheat spindle streak mosaic virus (WSSMV) (Zhang *et al*., 2005). The *Pm21* is located on the short arm of chromosome 6V in *H. villosa* and confers durable and broad‐spectrum resistance to *Bgt* (Chen *et al*., 1995). Previous studies have shown that *Stpk‐V* conferred broad‐spectrum powdery mildew resistance (Cao *et al*., 2011). Recently, an immediate‐early up‐regulated gene *CMPG1‐V* was documented contributing to powdery mildew resistance (Zhu *et al*., 2015). Here, we report the cloning of an L‐type *LecRK* from *H. villosa, LecRK‐V*, which was rapidly up‐regulated in response to *Bgt* inoculation and chitin treatment. Overexpressing *LecRK‐V* in transgenic wheat resulted in broad‐spectrum powdery mildew resistance.

## Results

### Cloning and sequence analysis of *LecRK‐V*

**Figure 1:**
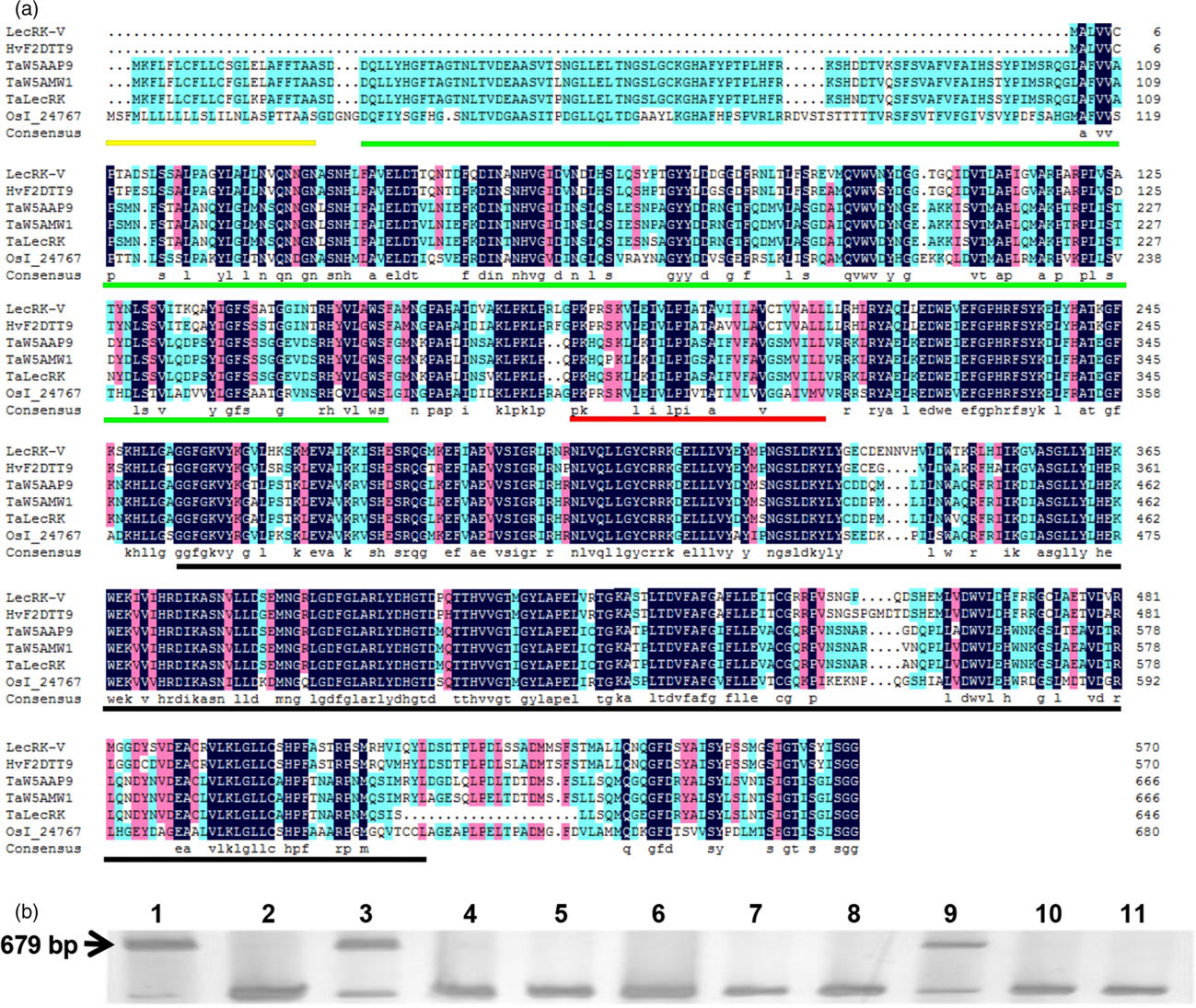
Sequence alignment, phylogenetic analysis and chromosome location of LecRK‐V and its homologues. (a) Alignment of LecRK‐V with its homologues in wheat, rice and barley. GenBank accession number of HvF2DTT9 is BAJ98510.1 was predicted from AK367307.1, OsI_24767 is EAZ02656.1. The protein sequences of wheat were obtained from UniProtKB: TaW5AAP9 (W5AAP9_WHEAT); TaW5AMW1 (W5AMW1_WHEAT) and TaLecRK/Ta‐W4ZXF8 (W4ZXF8_WHEAT) are included. The signal peptide was underlined as yellow line, the legume‐like lectin domain was underlined as green line, the serine/threonine kinase domain was underlined as black and transmembrane domain was underlined as red; (b) Chromosomal location of *LecRK‐V* by PCR using various genetic stocks. 1: *H. villosa* (2*n* = 2*x* = 14, genome VV); 2: *T. durum* (2*n* = 4*x* = 28, genome AABB); 3: *T. durum*‐*H. villosa* amphiploid (2*n* = 6*x *= 42, genome AABBVV); 4: wheat variety Chinese spring (2*n* = 6*x* = 42, genome AABBDD); 5 to 11: *T. aestivum*‐*H. villosa* addition lines (2*n* = 6*x* = 44, genome AABBDD plus 1V1V‐7V7V, each contains one pair of chromosomes 1V to 7V of *H. villosa* in the common wheat background). The arrow indicated the 679‐bp amplicon specific for *LecRK‐V*.

Degenerate primer pair, LecRK‐V‐D‐F and LecRK‐V‐D‐R, was designed according to the sequence of the conserved domain of *AtLPK1* (At4G02410), *AtLecRK‐VI.2* (AT5G01540), *NbLRK1* (AB247455) and *TaLRK10* (AF085168.1). A 675 bp sequence was isolated from *H. villosa* cDNA at 12 h and 24 h after *Bgt* inoculation (hai). Sequence analysis suggests it is an L‐type LecRK belonging to receptor‐like kinase (RLK). To clone the full‐length gene, the cloned sequence was used to search the NCBI database and a barley sequence (accession number:AK367307.1) encoding lectin receptor kinase was identified. Primer pair, LecRK‐V‐FL‐F and LecRK‐V‐FL‐R, was further designed according to AK367307.1 and used for cloning its homologues in *H. villosa*. A full‐length cDNA of 1716 bp corresponding to an L‐type LecRK was cloned and named as *LecRK‐V* (GenBank Accession: KY612459). The LecRK‐V has an extracellular L‐type lectin domain at the N‐terminus, a transmembrane domain and an intracellular serine/threonine kinase domain at the C‐terminus (Figure 1a). Its open reading frame (ORF) encodes a 572 amino acids protein with predicted molecular weight of 62.9 kDa and an isoelectric point of 7.49.

The chromosomal location of *LecRK‐V* was determined by amplification using DNA from *T. durum*‐*H. villosa* amphiploid (genome AABBVV) and a complete set of Chinese spring‐*H. villosa* alien addition lines (DA1V–7V). A 679‐bp product was amplified in *H. villosa*, the amphiploid and addition line DA5V, but not in Chinese spring and the remaining tested addition lines. Thus, the *LecRK‐V* was mapped to the chromosome 5V of *H. villosa* (Figure 1b).

By searching the IWGSC, EBI and NCBI databases, a total of 275 LecRKs were identified in the genome of wheat, barley, *T. urautu* and *Ae. tauschii*. Together with the LecRK‐V, a phylogenetic tree was constructed using AtFLS2 as outgroup. The 276 LecRKs could be classified into 11 different types, which were designated as LecRK‐I‐LecRK‐XI. There are 30, 48, 44, 8, 9, 14, 18, 27, 4, 40 and 34 LecRKs in each of LecRK‐I‐LecRK‐XI, respectively. The LecRK‐V was resided in LecRK‐XI (Figure S1, Table S2).

**Figure 2:**
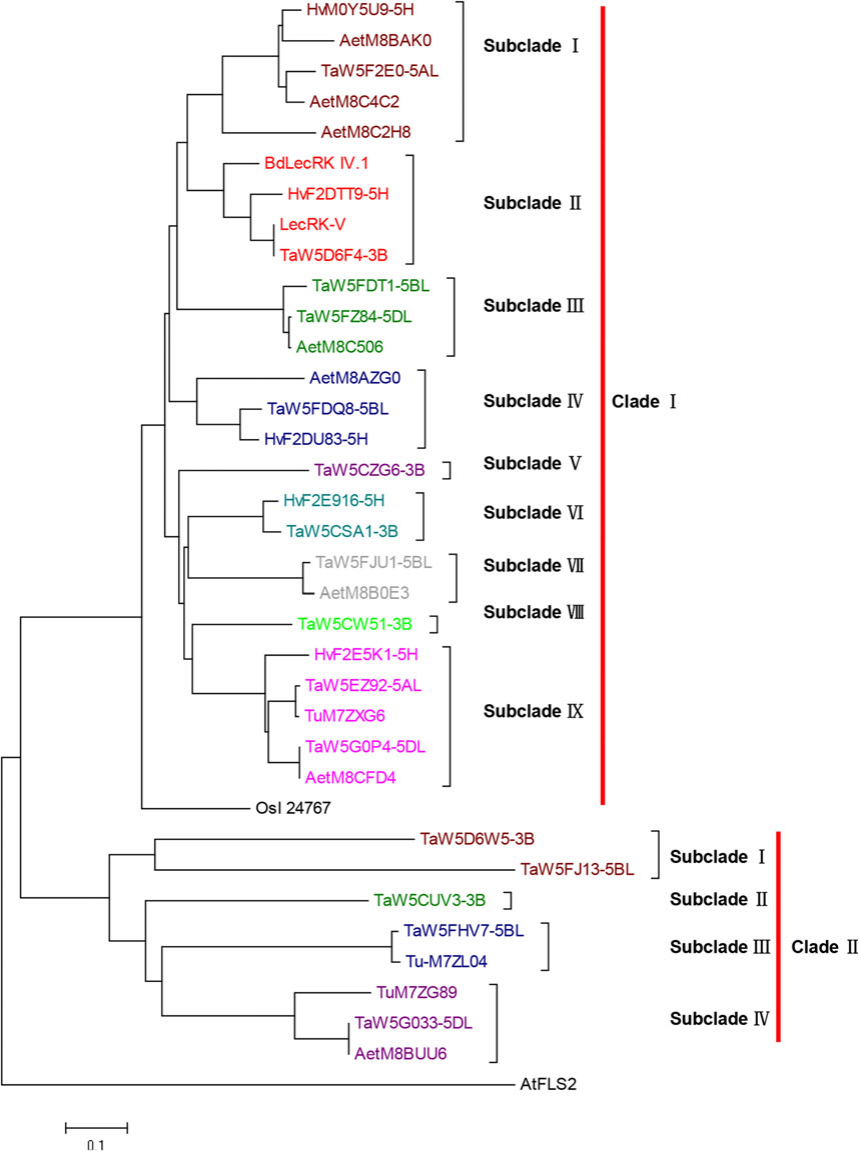
Phylogenetic tree of LecRK‐V and its homologues. GenBank accession numbers: AtFLS2 is NP_199445.1, OsI_24767 is EAZ02656.1, BdLecRK IV.1 is KQK20379.1. The protein sequences of Barley, wheat, *T. urautu* and *Ae. tauschii* were predicted from IWGSC database or obtained from UniProtKB (Table S2). The tree was generated by ClustalX1.83 analysis with the corrected full‐length LecRK‐V protein sequences using neighbour‐joining method (MEGA6.0 software). The bar beneath the dendrogram represents a distance of 0.1 changes per amino acid.

A total of 34 LecRK‐V homologues were identified from six grass species, including wheat, barley, *T. urautu*,* Ae. tauschii*, rice and *Brachypodium distachyon*. In barley, there were five LecRK homologues, and in wheat there were 16, in which 6 were on 3B, 2 were on 5A, 5 were on 5B and 3 were on 5D, respectively. There were 3 and 8 LecRK homologues in *T. urautu* and *Ae. tauschii*, respectively. Using the AtFLS2 as outgroup, the LecRK homologues phylogenetic tree contains two well‐defined branches, Clade I and Clade II. The Clade I can be divided into nine subclades, and the LecRK‐V was included in the subclade II, in which there were four members, including the homologues from chromosomes of common wheat 3B, barley 5HL and *B. distachyon* Bd1. The other wheat homologues of LecRK‐V were either located on chromosome 3B or homoeologous group 5 (Figure 2). BLASTn (http://blast.ncbi.nlm.nih.gov/blast.cgi) showed that *LecRK‐V* orthologs in *B. distachyon* (*BdLecRK,* KQK20379.1) and in rice (*OsI_24767*) were located on chromosomes Bd1 and R7, which are syntenic to wheat homoeologous group 5 and group 2, respectively. We further found that TaW5DAF4, the chromosome 3B orthologs of LecRK‐V, has two complete same copies.

### LecRK‐V is predominantly localized on the plasma membrane

**Figure 3:**
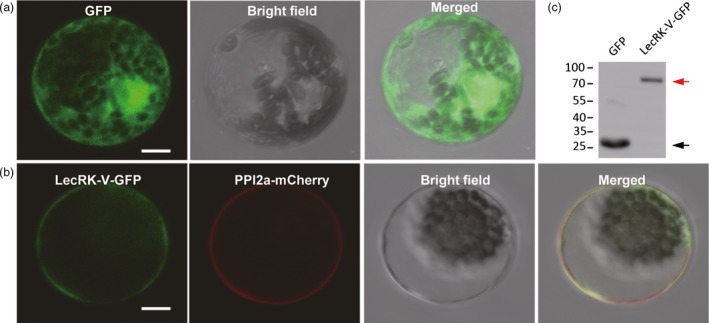
Subcellular localization of LecRK‐V. (a) The GFP signal was evenly distributed in the nucleus, cytoplasm and plasma membrane; (b) The LecRK‐V‐GFP fusion protein was overlapped with the PIP2a‐mCherry and localized on plasma membrane; Scale bar=10 μM; (c) GFP or LecRK‐V‐GFP were extracted from protoplasts and used for Western blotting using antibody anti‐GFP. The black and red arrows indicate the GFP and LecRK‐V‐GFP, respectively.

TMHMM software analysis predicted the present of transmembrane domain (190 to 212 amino acids) in LecRK‐V. The subcelluar localization of LecRK‐V was determined by co‐transforming the fusion vector 35S::*LecRK‐V*‐GFP and the plasma membrane marker protein vector PIP2α‐mCherry, into wheat protoplast. Compared with the evenly distribution of GFP fluorescence (Figure 3a), LecRK‐V‐GFP was localized on plasma membrane, which was confirmed by their overlapping with the PIP2α‐mCherry (Figure 3b). Detection of the 89 kDa band suggested that the intact LecRK‐V‐GFP fusion protein was expressed normally in the protoplasts (Figure 3c).

### *LecRK‐V* showed tissue‐specific expression pattern and was rapidly induced in *H. villosa* leaves in response to *Bgt* infection and chitin treatment

**Figure 4:**
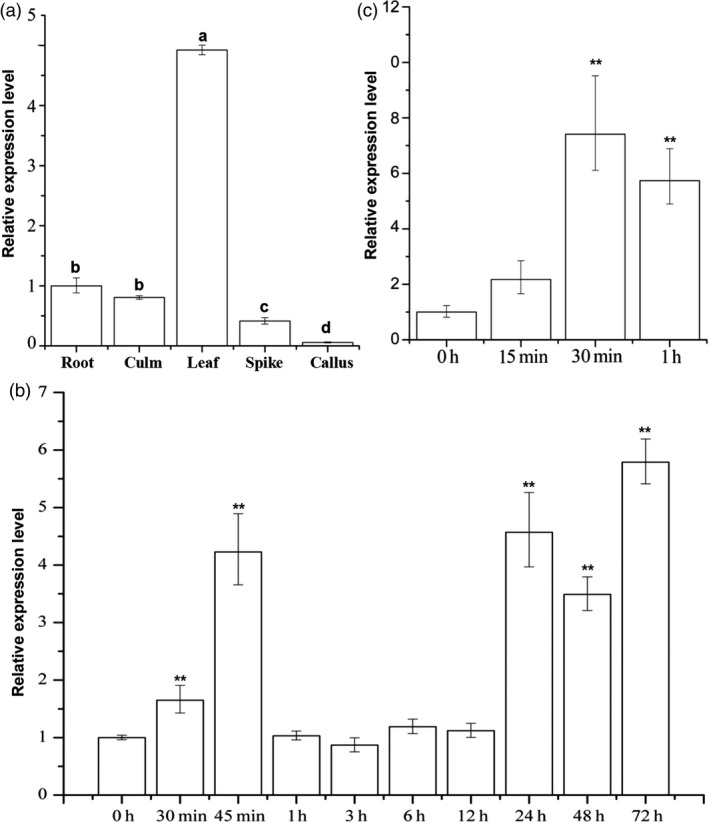
Expression profile of *LecRK‐V*. (a) Quantitative RT‐PCR (qRT‐PCR) of the relative *LecRK‐V* expression in different tissues of *H. villosa*. The qRT‐PCR values were normalized to those for *Tubulin*, and presented as fold changes relative to root; (b‐c) qRT‐PCR of *LecRK‐V* expression in the leaves of *H. villosa* in response to *Bgt* inoculation (b) and chitin treatment (c), The qRT‐PCR values were normalized to those for *Tubulin*, and presented as fold changes relative to *H. villosa* without *Bgt* inoculation (b) and chitin treatment (c), **P < 0.01.

The expression of *LecRK‐V* showed tissue specific in *H. villosa*. The transcript of *LecRK‐V* was more in leaves than those in roots, culms and spikes, and very low level in callus (Figure 4a). In *H. villosa* leaves, *LecRK‐V* was rapidly up‐regulated and reached a peak at 45 min after *Bgt* inoculation, which was about 4.25‐fold compared to noninoculated *H. villosa*. The *LecRK‐V* expression was decreased and back to 0.5‐ to 1.5‐ fold from 1 hai to 12 hai. From 24 hai to 72 hai, the *LecRK‐V* expression maintained a high expression level ranging from 3.15‐ to 4.33‐fold (Figure 4b).

The LecRK‐V was subcellularly localized on plasma membrane, indicating its possible role in sensing the PAMPs, such as chitin. We further tested the *LecRK‐V* expression pattern after chitin treatment. qRT‐PCR showed that *LecRK‐V* transcription reached a peak level at 30 min after chitin treatment, which is 15 min earlier than *Bgt* treatment, and was back to a relatively lower level at 1 h after treatment. This indicated *LecRK‐V* responded to chitin stimuli rapidly (Figure 4c).

### Transient and stable overexpression of *LecRK‐V* in Yangmai158 improved the powdery mildew resistance

**Figure 5:**
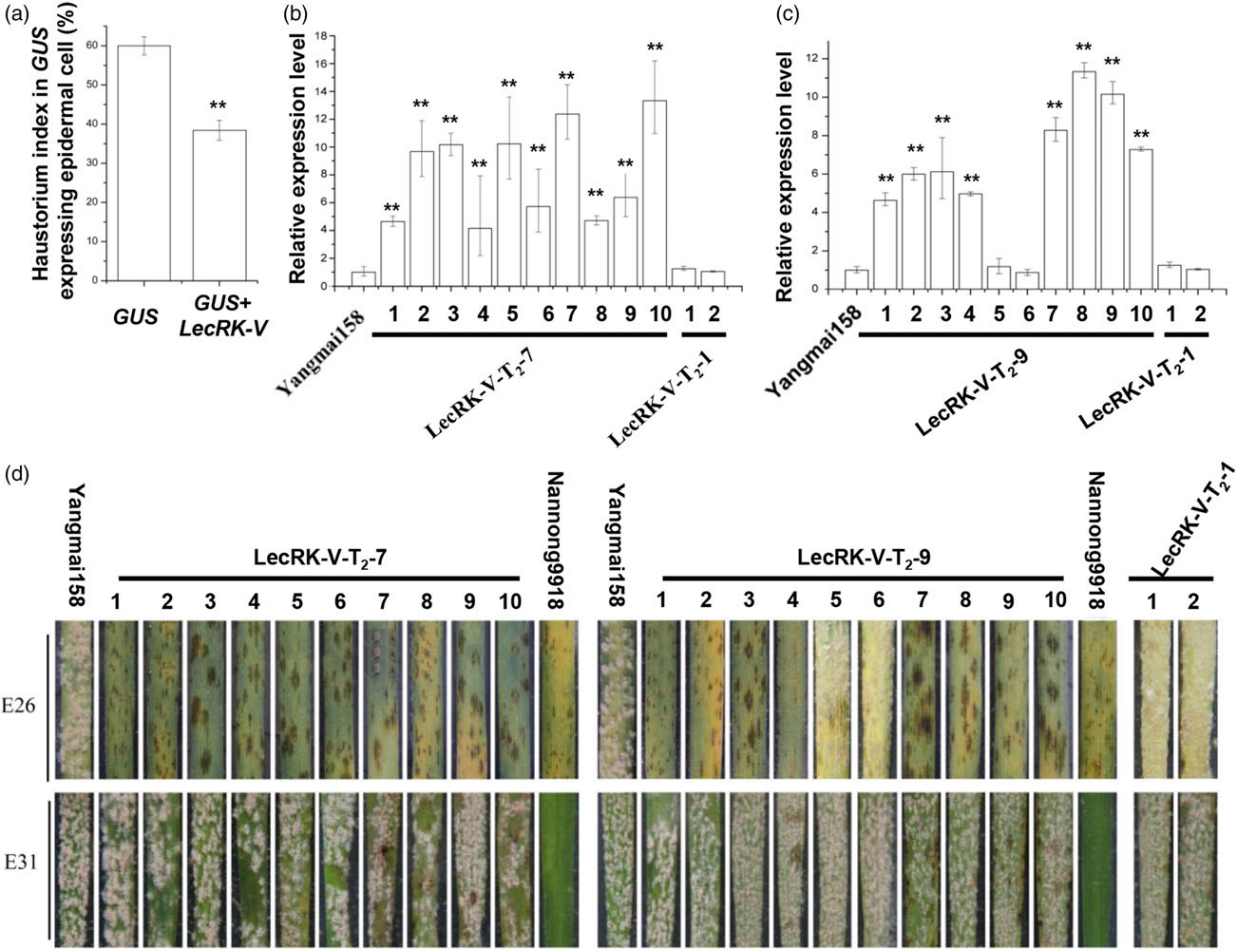
Functional analysis of *LecRK‐V* by transient single‐cell overexpression assay and transgenic approach. (a) Single‐cell transient overexpression assay by transforming *GUS* gene alone (control) and co‐transforming *GUS* and *LecRK‐V* in leaf epidermal cell of Yangmai158, *P < 0.01; (b–c) The *LecRK‐V* expression in the leaves of line LecRK‐V‐T_2_‐7 (b) and LecRK‐T_2_‐9 (c), The qRT‐PCR values were normalized to those for *Tubulin*, and presented as fold changes relative to Yangmai158, **P < 0.01; (d) Foliar parts of lines LecRK‐V‐T_2_‐7 and LecRK‐T_2_‐9 either inoculated with *Bgt* isolate E26 or E31. Photographs were taken 7dai.

An expression vector *pBI220‐LecRK‐V* was constructed by cloning *LecRK‐V* gene into *pBI220* driven by 2 × 35S promoter. Single‐cell transient overexpression assay was performed using Yangmai158, which is moderately susceptible to powdery mildew, as receptor. The haustorium index (HI) in the epidermal cells was used to measure the compatibility of wheat‐*Bgt* interaction. When transforming *pAHC25* carrying the *GUS* gene alone, the HI of Yangmai158 was 59.99%. While, when co‐transforming *GUS* with *pBI220‐LecRK‐V*, the HI decreased to 34.9%, which was considered as reaching a resistance level (Figure 5a). This indicated transient overexpression of *LecRK‐V* enhanced the powdery mildew resistance by preventing haustorial formation.

**Figure 6:**
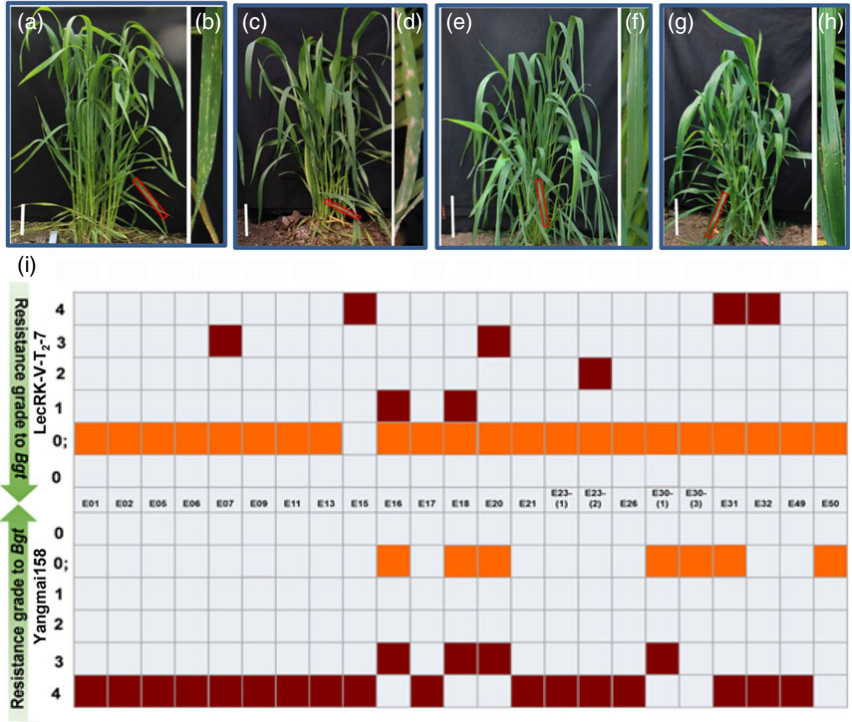
Powdery mildew resistance of Sumai 3 (a and b), Yangmai158 (c and d), the transgenic lines LecRK‐V‐T2‐7 (e and f) and LecRK‐V‐T2‐9 (g and h) in the field. The (b), (d), (f) and (h) showed enlarged views of the regions indicated by red boxes; (i) The evaluation of line LecRK‐V‐T_2_‐7 for resistance to 23 *Bgt* isolates at the seedling stage. Ordinate indicates the infection types (ITs) to various isolates which is shown as different coloured boxes. The grades 0, 0; and grades1‐2 which are considered as resistance level are shown as orange, and grades 3–4 which are considered as susceptible level are shown as dark red.

The vectors *pBI220‐LecRK‐V* and *pAHC20* (carrying *Bar* gene as a selection marker) were co‐transformed into Yangmai158 by particle bombardment into calli from 2000 wheat young embryos. After three rounds of selection, a total of 280 regenerated T_0_ plants were obtained. By PCR, nine positive transgenic plants were identified (Figure S2a), and all showed high level of powdery mildew resistance when infected by *Bgt* mixture inoculums collected from Nanjing, Jiangsu province (Figure S2e). qRT‐PCR showed that *LecRK‐V* was overexpressed in seven plants compared to those in Yangmai158 and the negative transgenic plant (Figure S2d). LecRK‐V‐T_0_‐37‐1 and LecRK‐V‐T_0_‐44‐4, which showed 9.35‐ and 15.32‐fold higher *LecRK* expression, were selected for further analysis. Their derived T_1_ lines, LecRK‐V‐T_1_‐7 and LecRK‐V‐T_1_‐9 were identified as positive lines (Figure S3a and b). They both showed enhanced resistance to *Bgt* mixture (Figure S3c and d), and their *LecRK* transcripts accumulated 2.12‐ to 8.27‐fold and 3.21‐ to 10.37‐fold higher than the receptor Yangmai158, respectively (Figure S3e and f). At T_2_ generation, ten and eight positive plants from LecRK‐V‐T_2_‐7 and LecRK‐V‐T_2_‐9 were identified by PCR (Figure S2b and c), and their *LecRK* transcripts accumulated 4.15‐ to 13.33‐fold higher than Yangmai158 (Figure 5b and c). The detached leaves were inoculated with two *Bgt* isolates, E26 and E31. Wheat variety Nannong 9918 (containing the *Pm21* gene) was resistant to both isolates. When inoculated by E26, the positive plants showed high resistance, while when inoculated by E31, they showed hypersensitive response at early infection but finally were susceptible (Figure 5d). The two transgenic lines both showed high resistance to *Bgt* mixture in the field (Figure 6a–h).

The line LecRK‐V‐T_2_‐7 was further evaluated for its resistance to 23 different *Bgt* isolates at seedling stage. LecRK‐V‐T_2_‐7 exhibited strong HR to all isolates except E15 and was resistant to 18 isolates (except E07, E15, E20, E31 and E32), while Yangmai158 exhibited HR to 6 isolates (including E16, E18, E20, E30‐(1), E30‐(3) and E50) and was only resistant to E30‐(3) and E50 (Figure 6i). These results indicated that *LecRK‐V* conferred broad‐spectrum resistance to powdery mildew and HR contributed to the incompatible interaction between the transgenic wheat and *Bgt*. Line LecRK‐V‐T_3_‐2 (derived from LecRK‐V‐T_2_‐7) exhibits enhanced powdery mildew resistance (Figure S4a). Southern blot detected two additional copies of *LecRK‐V* in this line (Figure S4b), indicating the gene has been integrated into the wheat genome.

### The ROS and SA pathways may be associated with the powdery mildew resistance mediated by *LecRK‐V*

**Figure 7:**
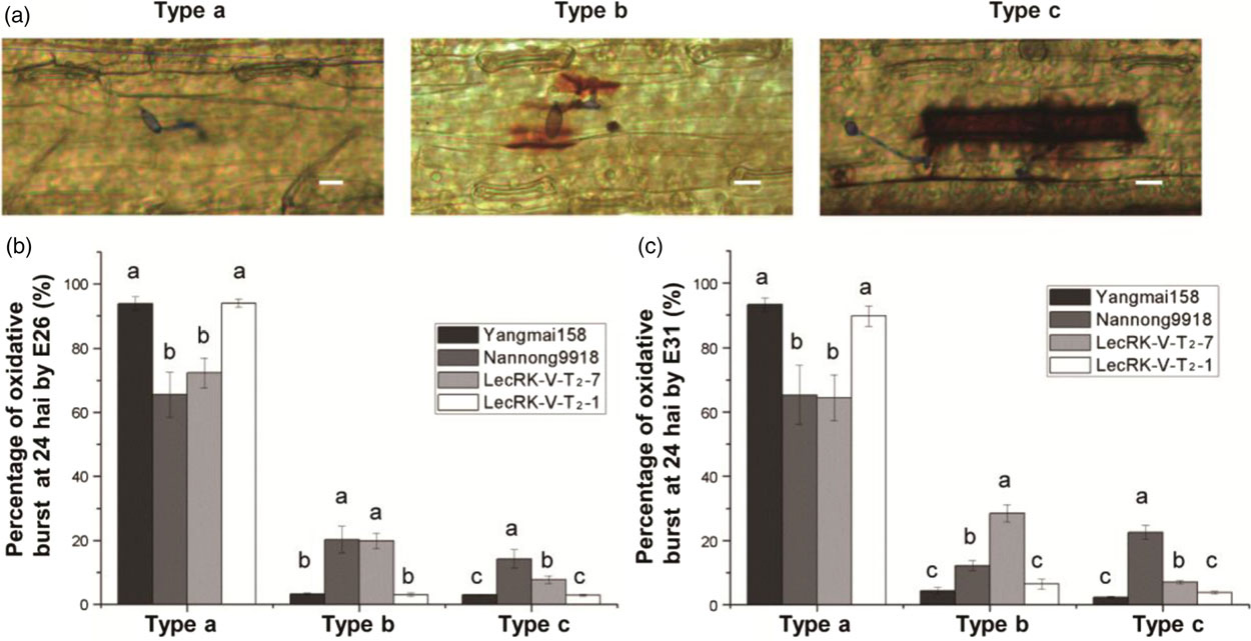
Comparison of the amount of ROS accumulation in Yangmai158, Nannong9918, positive transgenic line LecRK‐T_2_‐7 and negative transgenic line LecRK‐V‐T_2_‐1. (a) Three types of DAB stained cell. Type a: no observed oxidative burst, Type b: oxidative burst only at *Bgt* infection site; Type c: oxidative burst distributed in whole cell. Bar = 10 μm. (b‐c): Comparison of the percentage of the three types of DAB stained cell after inoculation with isolates E26 (b) and E31(c).

Nannong 9918 is highly resistant to both E26 and E31; however, HR was only observed when inoculated with E26. Interestingly, hypersensitive response (HR) was observed in the transgenic plants when inoculated by either E26 or E31; however, the HR was much stronger in the interaction with E26 than E31 (Figure 5d). The HR may contribute to the prevention or delay of further infection of E26 or E31. Two negative transgenic plants derived from line LecRK‐V‐T_2_‐9 and the negative line LecRK‐V‐T_2_‐1 all were highly susceptible to both E26 and E31, and similar to the receptor Yangmai158, they both had less HR. The H_2_O_2_ accumulation at infection sites in epidermal cells was compared at 24 hai by E26 and E31 of the positive transgenic line LecRK‐V‐T_2_‐7, negative transgenic line LecRK‐V‐T_2_‐1, Yangmai158 and Nannong9918. Base on the observation, the infected cells can be classified into three different types: Type a (no observed oxidative burst), Type b (oxidative burst only at *Bgt* infection site) and Type c (oxidative burst distributed in whole cell) (Figure 7a). After E26 and E31 inoculation, most cells at the infection sites were not or only slightly stained in Yangmai158 and LecRK‐V‐T_2_‐1. After E26 inoculation, the percentages of Type b and Type c cells were significantly higher in Nannong9918 (20.27% and 14.26%, respectively) and line LecRK‐V‐T_2_‐7 (19.9% and 7.73%, respectively) than in Yangmai158 (3.25% and 2.99%, respectively) and negative line LecRK‐V‐T_2_‐1 (3.07% and 2.92%, respectively) at infection sites (Figure 7b). After E31 inoculation, compared to Yangmai158 (4.37%) and line LecRK‐V‐T_2_‐1(6.47%), the percentage of Type b cells was significantly increased in LecRK‐V‐T_2_‐7 (28.48%), even higher than that in Nannong9918 (12.21%). Accordingly, the Type c cells were also significantly increased in LecRK‐V‐T_2_‐7 (7.08%) compared to Yangmai158 (2.38%) and LecRK‐V‐T_2_‐1 (3.79%), but lower than in Nannong9918 (22.52%) at infection sites (Figure 7c). Quantitative analysis showed significant increase of H_2_O_2_ accumulation in the *LecRK‐V* overexpressing transgenic plants when infected by E26 or E31 (Figure 7b and c).

**Figure 8:**
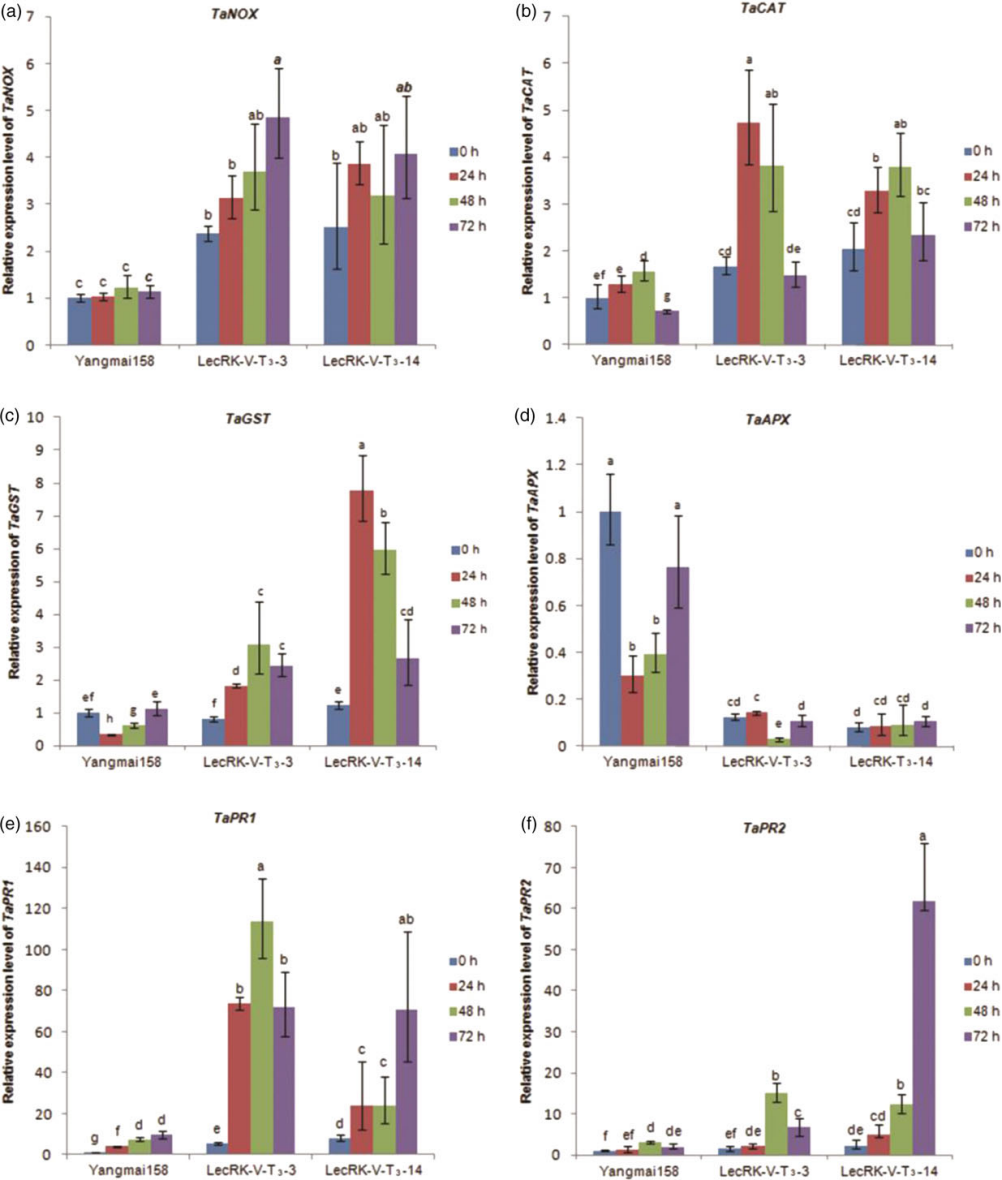
Relative expression of *TaNOX*(a), *TaCAT*(b), *TaGST*(c), *TaAPX*(d), *TaPR1*(e), *TaPR2*(f) in lines LecRK‐V‐T_3_–3, LecRK‐V‐T_3_–14 and Yangmai158 after *Bgt* inoculation. The qRT‐PCR values were normalized to those for *Tubulin* and presented as fold changes relative to Yangmai158 without treatment. Three independent experiments were performed, and similar results were obtained; the standard deviation (*n* = 3) is represented by error bars. Different letters indicate statistically significant differences (*P* < 0.01, one‐way ANOVA).

The expression of ROS generating/scavenging genes, including *TaNOX* (GenBank accession: AY561153.1), *TaCAT* (GenBank accession: HM989895.1) and *TaGST* (GenBank accession: AJ441055), *TaAPX* (GenBank accession: EF555121.1), was further investigated in two transgenic wheat and Yangmai158. The results showed that compared to Yangmai158, the expression levels of *TaNOX* (Figure 8a) and *TaCAT* (Figure 8b) were two fold increased in the transgenic plants before *Bgt* inoculation. The expression levels of *TaNOX* (Figure 8a), *TaCAT* (Figure 8b) and *TaGST* (Figure 8c) were significantly increased in the transgenic plants at 24, 48 and 96 hpi. However, *TaAPX* exhibited lower transcription in the transgenic plants both before and after *Bgt* inoculation (Figure 8d). These results indicated that transformation of *LecRK–V* into Yangmai158 changed of the expression of ROS generating/scavenging genes in response to *Bgt* infection, further confirmed the involving of the ROS pathway in the *LecRK–V*‐mediated resistance.

Expression of PR genes related to the SA phytohormone pathway was further analysed in the two T_3_ transgenic lines LecRK‐V‐T_3_–3 and LecRK‐V‐T_3_–14 (derived from LecRK‐V‐T_2_‐7 and LecRK‐V‐T_2_‐9, respectively). The expression levels of genes *TaPR1* (GenBank accession: AF384143) and *TaPR2* (GenBank accession: DQ090946) were higher in transgenic plants than in Yangmai158 before and after *Bgt* treatment (Figure 8e and f), indicating that the SA pathway may participate in the defence response of transgenic plants against *Bgt* infection.

### Knockdown of *LecRK‐V* by BSMV‐mediate Virus Induced Gene Silencing (VIGS) in *T. durum*‐*H. villosa* amphiploid could not compromise the powdery mildew resistance

Ten days after BSMV inoculations on leaves of *T. durum*‐*H. villosa* amphiploid, mild chlorotic mosaic symptom was observed, indicating the success of the VIGS system. qRT‐PCR showed that the *LecRK‐V* expression was significantly down‐regulated when inoculated with BSMV:*LecRK‐V* (Figure S5a), indicating successful silencing of *LecRK‐V*. Leaves from Mock, BSMV: *PDS* and BSMV:*LecRK‐V* treatments were inoculated with E26 and E31. At 7 dai, no disease symptom was observed on any leaf no matter the *LecRK‐V* was silenced or not when inoculated by E26 (Figure S5b) or E31 (Figure S5c). Further observation of appressorial germ tube (AGT) and appressorium penetration peg (app) under the microscope showed that the growth and development of E26 (Figure S5d‐f) and E31 (Figure S5g‐i) had no difference among the three treatments.

## Discussion

Plant L‐type LecRKs, which are characterized with an extracellular soluble legume lectins domain including a specific carbohydrate‐protein binding site, are conserved present in higher plants (Bouwmeester and Govers, 2009). To date, 45 LecRKs and 170 LecRKs were identified in *Arabidopsis* and rice, respectively. To explore the role of LecRKs in wheat, we searched the L‐type LecRKs in sequenced wheat and its relatives, and 81, 87, 44 and 63 sequences of L‐type LecRKs were obtained from *T. aestivum*,* H. vulgare*,* T. urartu* and *Ae. tauschii*, respectively. Considering about three fold larger genome size of hexaploid wheat than other three diploid *Triticea* species, the number of the identified L‐type LecRKs in common wheat was relatively low. In addition, the function of most of these *LecRKs* remains elusive, except *TaLRK10* (GenBank: AAC49629.1). *TaLRK10* was characterized as a candidate of *Lr10* (Feuillet *et al*., 1997).

To investigate the role of L‐type *LecRKs* in powdery mildew resistance conferred by *H. villosa*,* LecRK‐V* was cloned from *H. villosa* and was localized on chromosome 5V. Even though we identified four LecRKs were present on homoeologous groups 5, that is chromosomes 5A (5AL, cM = 144.93), 5B (5BL, cM = 130.69), 5D (5DL, cM = 144.93) and barley 5H (5H, cM = 139.23), but they are not in same collinearity region. Interestingly, the closest homologue of *LecRK‐V*, TaW5DAF4, was present on wheat chromosome 3B, in which there are two copies having 100% sequence identities. However, we failed to find the *LecRK‐V* homologues on chromosomes 3A and 3D. One possibility is that the short‐read‐based assembly sequences are still fragmented, and a proportion of genes have not been assembled and annotated (Choulet *et al*., 2014; Jia *et al*., 2013; Ling *et al*., 2013; Mayer *et al*., 2014). In diploid barley, we predicted 81 LecRKs, while in hexaploid wheat, only 87 LecRKs were predicted, far less than expected. Another possibility is that the 3B is the largest chromosome (with a genome size of 1 Gb), during polyploidization and evolution of wheat, the 3B exhibits high rate of intrachromosomal duplication and may obtain new genes (Choulet *et al*., 2014).

LecRK‐V contains an extracellular L‐type lectin domain for carbohydrate binding at N‐terminus. It has been reported that the interaction between lectin and carbohydrate plays an important role in immunity response. In mammalian, lectin–glycan interactions regulate cell death during physiologic and pathologic settings (Lichtenstein and Rabinovich, 2013). The lectin domain was speculated to recognize to diverse pathogens by interaction with PAMPs or HAMPs (host‐associated molecular patterns), such as oligosaccharides, PGNs and peptides in plant innate immunity. Glycans such as chitin and β‐glucan are major cell wall components of plant fungal pathogen. Plant LysM domain containing proteins, that is OsCEBiP, OsLYP4, OsLYP6 and AtCERK1, have been reported to be the receptor of chitin (Kaku *et al*., 2006; Liu *et al*., 2012; Miya *et al*., 2007). OsLYP4 and OsLYP6 can physically bind to PGNs (Liu *et al*., 2012). NbLRK1, the PRR for the INF1 elicitor, and CaMBL1, the receptor for mannose N‐glycans elicitor, was reported to positively regulate disease resistance (Hwang and Hwang, 2011; Kanzaki *et al*., 2008). LecRK‐V was located on plasma membrane in wheat protoplast, its extracellular domain contains a preserved hydrophobic cavity site and sugar‐binding residues; thus, LecRK‐V is presumed to be an ideal candidate for perceive various fungal cell wall integrity. *LecRK‐V* rapidly responded to both *Bgt* and chitin treatments, so we speculate that *LecRK‐V* may mediate wheat powdery mildew resistance at the period of PTI. We also observed the present of the second expression peak at 24 h after chitin treatment. This may be induced by the secondary infection, because the haustorium formed after 18 hai and the secondary hyphae emerged at around 24 hai.

Most of the identified positive transgenic lines showed overexpression of *LecRK‐V* and enhanced powdery mildew resistance. Two lines showed stable resistance to E26 but were susceptible to E31, and the HR in the interaction of the transgenic plant with the two isolates was also different. This provides an ideal system for comparing the compatible and incompatible interactions between *LecRK‐V* transgenic wheat and *Bgt*.

In plant, ROS is an important signaling molecular, involved in signal propagation in numerous pathways and its role in defence has been extensively summarized. Various stimuli, including pathogen inoculation and pathogen elicitor treatment, triggers oxidative bursts that occur at early pathogen infection stages and induce HR later (Tenhaken *et al*., 1995). In Arabidopsis, the rapid occurrence of the HR was characterized as the main difference in compatible and incompatible interactions between the *Peronospora parasitica* isolate Emoy2 and *Arabidopsis thaliana* accessions (Soylu *et al*., 2004). Upon flg22 treatment, the immune receptor FLS2 complex activates BIK1 and PBL1 which directly phosphorylate RbohD at S39 and Ser343 to enhance ROS generation and then to orchestrate antimicrobial defences (Li *et al*., 2014). Overexpression of LecRK‐I.9 in Arabidopsis leads to HR and enhanced resistance to *P. brassicae* isolate CBS686.95 that is compatible to Col‐0 (Bouwmeester *et al*., 2011). Moreover, the T‐DNA insertions mutant *lecrk‐I.9* shows gain of susceptibility towards *P. brassicae* isolate HH that is incompatible to Col‐0 (Bouwmeester *et al*., 2011). In rice, constitute expression of a GTPase OsRAC1, an activator of plant NADPH oxidase, enhanced ROS production, caused HR‐like response and increased resistance against a virulent race of bacterial blight (Ono *et al*., 2001). NbLRK1, a lectin‐like receptor kinase protein of *N. benthamiana*, interacts with *Phytophthora infestans* INF1 elicitin and mediates INF1‐induced cell death. NbLRK1 is a component of the *N. benthamiana* protein complex that recognizes INF1 elicitor and transduces the HR signal (Kanzaki *et al*., 2008). In our study, significantly increased ROS accumulation and strong HR were observed in *LecRK‐V* transgenic wheat either by E26 or E31 inoculation, compared to Yangmai158 and the negative transgenic wheat. Consistently, dynamic changes were detected for the expression levels of ROS generating/scavenging genes and marker genes of the SA pathway, indicating that ROS and SA pathways may contribute to ROS accumulation and associate with the powdery mildew resistance mediated by *LecRK‐V*. We speculate that higher ROS accumulation and PR gene expression in *LecRK‐V* transgenic wheat contribute to its strong HR and resistance to E26, while in E31, there may exist an uncertain effector to overcome this resistance mediated by *LecRK‐V*.

*Pm21* gene is located on the short arm of chromosome 6V in *H. villosa* and confers broad‐spectrum powdery mildew resistance (Cao *et al*., 2011). When silenced *LecRK‐V* in *T. durum*‐*H. villosa* amphiploid, we failed to observe any symptom for the change of disease resistance level, both for the disease phenotype and *Bgt* development. *T. durum*‐*H. villosa* amphiploid contains the *Pm21* gene. We suspect that resistance conferred by *LecRK‐V* was independent of the *Pm21‐*mediated resistance pathway. Phylogenetic analysis demonstrated the different members of *LecRKs* were existed in *T. aestivum*. Thus, gene redundancy may be another possible explanation.

## Experimental procedures

### Plant material

*H*. *villosa* (introduced from Cambridge Botanical Garden, United Kingdom, Accession No. 91c43), *T*.* durum*‐*H*. *villosa* amphiploid [developed by the Cytogenetics Institute of Nanjing Agricultural University (CINAU) (Accession No. NAU201)], Yangmai158 (receptor wheat variety for genetic transformation), Nannong9918 (Wheat‐*H*. *villosa* translocation line T6VS·6AL developed by CINAU. A**c**cession No. NAU405) and Sumai 3 (for producing fresh *Bgt* inoculums) were used in this study. All the accession numbers and seeds were provided by CINAU. E26, E31and mixed *Bgt* isolates were maintained on susceptible variety Sumai 3 seedlings in a spore‐proof greenhouse under 14‐h light/10‐h dark (24/18 °C, 70% humidity) regime.

### Cloning of LecRK‐V

The degenerate primer pairs LecRK‐D‐F and LecRK‐D‐R for isolating lectin receptor kinase gene from *H. villosa* were designed according to the sequence of the conserved domain of *AtLPK1* (At4G02410), *AtLecRK‐VI.2* (AT5G01540), *NbLRK1* (AB247455) and *TaLRK10* (AF085168.1). *LecRK‐V* was isolated from *H. villosa* cDNA at 12 h, 24 h after *Bgt* inoculation. PCR was performed at 95 °C for 5 min, followed by 30 cycles of 94 °C for 15 s, 56 °C for 1 min and 72 °C for 2 min and then by 10 min at 72 °C. Phanta Max Super‐Fidelity DNA polymerase (Vazyme, Nanjing, China) was used for amplification.

### Expression vector construction and single‐cell transient overexpression assay

The ORF fragment of the LecRK‐V was amplified using primer pair LecRK‐V‐F/LecRK‐V‐R (Table S1). In the recombinant vector *pBI‐220‐LecRK‐V*, the *LecRK‐V* gene was placed under the control of the 2 × 35S promoter and followed by the NOS terminator sequence. The single‐cell transient expression assay was performed as described (Shirasu *et al*., 1999). The reporter plasmid *pWMB002* containing β‐glucuronidase (*GUS*) gene and the plasmid *pBI‐220‐LecRK‐V* were mixed before coating of the particles (molar ratio of 1:1; 1 μg of total DNA). The bombarded leaves were transferred to 1% agar plates supplemented with 85 μm benzimidazole and incubated at 22 °C for 8 h before high‐density inoculation with single *Bgt* spores. Leaves were GUS stained to identify *LecRK‐V* transformed cells at 48 hai. The haustorium index (HI, percentage of GUS‐staining cells with haustoria in the total GUS‐staining cells attacked by *Bgt*) is indicated by the mean of three independent experiments, each contributing at least 40 interactions.

### Wheat transformation

The herbicide tolerance gene *Bar*, which is driven by maize Ubiquitin promoter and followed by the NOS terminator sequence, was used as a selectable marker gene in vector *pAHC20*. The vectors *pBI‐220‐LecRK‐V* and *pAHC20* were co‐transformed into wheat calli by particle bombardment. The calli were cultured from immature embryos of wheat variety Yangmai158. The regenerated plants were produced as described by Xing *et al*. (2008). For screening of the positive transgenic plants, specific combined primer pair CAMV35S‐F/LecRK‐V‐SP‐R (Table S1) was designed to amplify the sequence covering the 2 × 35S promoter and part of the *LecRK‐V*. The transgene was identified in successive T_0_ to T_2_ generations. PCR was performed at 94 °C for 3 min; 35 cycles of 94 °C for 30 s, 57 °C for 40 s, and 72 °C for 1 min; followed by 10 min at 72 °C. The PCR products were separated on 1% agarose gels.

### Southern blot analysis

Genomic DNA was isolated from leaves of the transgenic line and Yangmai158 using Plant Genomic DNA Kit (No. DR14‐100T, ZoonBio, Nanjing, China). Southern blot was performed as described by Stein *et al*. (2001). Ten microlitres genomic DNA was digested with restriction enzyme *Hind*III, and the digested DNA was fractionated on a 0.8% agarose gel and transferred onto a Nylon Hybond‐N^+^ membrane (No.RPN303B, Amersham, Sweden) with a membrane transfer instrument (Model 785, Bio‐Rad). The probe L‐1 was labelled with Digoxigenin using PCR DIG Probe Synthesis Kit (No. 11636090910, Roche, Germany), and *pBI‐220‐LecRK‐V* was used as template for PCR using the primer pair L‐1‐F (5′CGAGGCAA‐ GGGATGAAGG3′) and L‐1‐R (5′ CGAACACGTCTGTGAGGGTC 3′). PCR was performed with the following parameters: 35 cycles of 30 s at 94 °C, 30 s at 60 °C and 30 s at 72 °C. The prepared probe L‐1 was used to hybridize to the membrane, and the hybridization and detection were performed according to the instructions for the DIG High Prime DNA Labeling and Detection Starter Kit II (No. 11585614910, Roche, Germany).

### Evaluation of powdery mildew resistance

Powdery mildew resistance of the transgenic plants was evaluated by inoculation with *Bgt*; Yangmai158 and Nannong9918 were used as susceptible and resistance control, respectively. For plant grown in an incubator, the first leaves at second leaf stage and for plants grown in the field the fourth leaves at fifth leaf stage were evaluated by inoculation with *Bgt*. The detached leaves from difference generation transgenic line, Yangmai158 and Nannong9918 were put on 0.6% agar plates supplemented with 85 μm benzimidazole and inoculated with fresh single *Bgt* isolation or *Bgt* Mix, incubated at 20 °C with 16‐h light per day and 80% relative humidity, and scored after 7 days. After powdery mildew evaluation at seedling stage, the plants in the field were inoculated with *Bgt* Mix for evaluation of powdery mildew resistance at adult stage. Twenty‐three *Bgt* isolates, kindly provided by Prof. Yilin Zhou (Institute of Plant Protection, Chinese Academy of Agricultural Sciences), were used to evaluate the powdery mildew resistance of T_2_ transgenic plants at seedling stage.

### Vector construction and subcellular localization of *LecRK‐V*

*Bam*H I and *Nhe* I sites were added to the 5ʹ and 3ʹ ends of the full‐length ORF of *LecRK‐V*, respectively. PCR products of *LecRK‐V* by primer pair LecRK‐V‐CL‐F/R (Table S1) and the vector *pAN580* were digested by *Bam*H I and *Nhe* I, and the fragments were ligated to produce the fusion gene expression vector *p35S::GFP‐LecRK‐V*. Constructs were transformed into the mesophyll protoplasts prepared from Yangmai158. Plasmid DNA (1.5 μg/μL) of each construct was mixed with PIP2α‐mCherry fused marker protein (1.5 μg/μL), and 20 μL DNA was used to transform 200 μL of protoplasts derived from five‐ to 7‐day‐old plants. The GFP/mCherry signals were assessed by confocal imaging 16–20 h after transformation. For imaging, a LSM780 (Carl Zeiss, Jena, Germany) confocal microscope was used with the following settings: GFP excitation, 488 nm; emission band pass, 505 to 520 nm; RFP excitation, 543 nm; and emission band pass, 560 to 615 nm.

### Gene expression analysis

*LecRK‐V* expression level was analysed by qRT‐PCR under *Bgt* treatment in *H. villosa*. *H. villosa* plants were grown at 22–25 °C with a photoperiod of 12 h. Plants were inoculated with a mixture of *Bgt* (collected from Jiangsu province, China) when they reached the second leaf stage. Leaves were sampled before treatment and at 30 min, 45 min, 1 h, 3 h, 6 h, 12 h, 24 h, 48 h and 72 h after inoculation (hai), and three biological replicates were used. To test expression profile of *LecRK‐V* in response to chitin treatment, *H. villosa* were treated with 100 μg/mL insoluble chitin (No. C7170, Sigma‐Aldrich, US). Leaves were sampled before treatment and at 15 min, 30 min, 1 h after treatment. Three biological replicates were used. For the transcript levels of *LecRK‐V* genes in different tissues of *H. villosa*, the roots, culms, leaves and spikelets were sampled at 20 day after flowering, and callus was sampled at 8 day after dedifferentiation in the SD2 medium. To evaluate expression of *LecRK‐V* and genes related to SA and ROS pathways, plants were inoculated with a *Bgt* mixture (collected from Jiangsu province, China) when they reached the second leaf stage, and the leaves were sampled before treatment and at 24 h, 48 h and 72 h after inoculation (hai).

All the RNA was isolated using Trizol reagent (Invitrogen, US) according to the manufacturer's instructions. First‐strand cDNA was synthesized from 1 μg total RNA using HiScript Q RT SuperMix for qPCR (Vazyme, Nanjing, China). The *Tubulin* (Table S1) was used as the internal control for normalization. Primer pairs for *LecRK‐V* and genes related to SA and ROS pathways are shown in Table S1. qRT‐PCR was performed with AceQ qPCR SYBR Green Master Mix (Vazyme, Nanjing, China) using PCR LightCycler 480 (Roche, Rotkreuz, Switzerland), where reactions were subjected to the following program: 95 °C for 5 min, 41 cycles of 95 °C for 10 s, and 60 °C for 31 s. For each sample, the *Ct* value of each target gene was normalized to the *Ct* value of the *Tubulin* gene. The relative value of gene expression was derived from 2^–▵▵CT^. Statistical analysis of the experimental data was conducted by ANOVA (one tailed) with the SPSS 10 program (SAS Institute Inc., Cary, NC).

### Functional analysis of *LecRK‐V* by VIGS

The 251‐bp residues of *LecRK‐V* were amplified by primer pair VIGS‐LecRK‐V‐F/R (Table S1) and were reversely inserted into BSMV:γ vector to produce the recombinant vector BSMV:*LecRK‐V*. Using the system established by Wang *et al*. (2010) with the following steps: At 10–12 day after virus inoculation, the fourth leaves were treated with *Bgt*. After 7 day, leaves were fixed, bleached and stained with Commassie blue for observation of fungal development in bright field under Olympus BX41 microscope. Ten leaves per sample were observed to evaluate the effect of gene silencing. The fifth leaves challenged with BSMV:*LecRK‐V* and BSMV:PDS were also used to check the expression level of *LecRK‐V* on the Roche PCR 480 sequence detection system using AceQ qPCR SYBR Green Master Mix (Vazyme, Nanjing, China).

### H_2_O_2_ detection by DAB staining

The first leaves were cut from Yangmai158 and T_2_ generation transgenic plants at 24hai of *Bgt*. H_2_O_2_ detection was performed by *in situ* histochemical staining using 3,3′‐diaminobenzidine as described (Thordal‐Christensen *et al*., 1997).

## Supporting information


**Figure S1.** The phylogenetic tree of 276 LecRKs from wheat, barley, *T. urautu, Ae. tauchii* and *H. villosa*.
**Figure S2.** Functional analysis of *LecRK‐V* transgenic wheat.
**Figure S3.** Functional analysis of *LecRK‐V* transgenic wheat at T_1_ generation.
**Figure S4.** Southern blot of transgenic line LecRK‐V‐T_3_–2
**Figure S5.** VIGS of *LecRK‐V* in *T. durum*‐*H. villosa* amphiploid.
**Table S1.** Information of the primer pairs used in this study.
**Table S2.** The 275 LecRKs identified in wheat, barley, *T. urautu* and *Ae. tauschii*.
