# Supplementary material for: LecRK‐V, an L‐type lectin receptor kinase in Haynaldia villosa, plays positive role in resistance to wheat powdery mildew
Source: Plant Biotechnol J. 2017 Aug 1;16(1):50–62. doi: 10.1111/pbi.12748 (PMC5811777; doi:10.1111/pbi.12748)
Supplement: Supplementary file 1 — Figure S1. The phylogenetic tree of 276 LecRKs from wheat, barley, T. urautu, Ae. tauchii and H. villosa. Figure S2. Functional analysis of LecRK‐V transgenic wheat. Figure S3. Functional analysis of LecRK‐V transgenic wheat at T1 generation. Figure S4. Southern blot of transgenic line LecRK‐V‐T3–2 Figure S5. VIGS of LecRK‐V in T. durum‐H. villosa amphiploid. Table S1. Information of the primer pairs used in this study. Table S2. The 275 LecRKs identified in wheat, barley, T. urautu and Ae. tauschii. [file PBI-16-50-s001.doc]

SUPPORTING INFORMATION


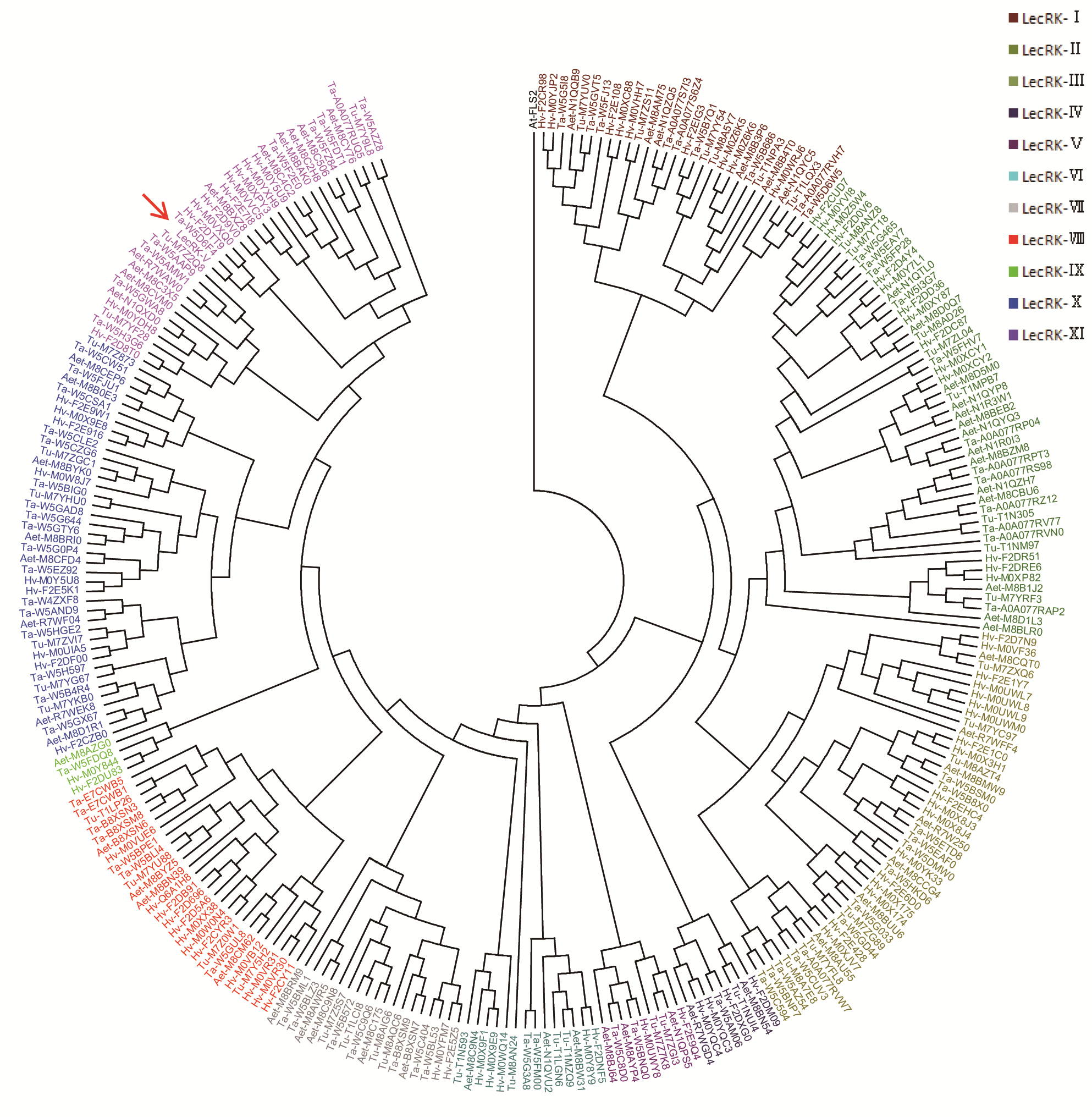


**Figure S1** The phylogenetic tree of 276 LecRKs from wheat, barley, *T. urautu,* *Ae. tauchii* and *H. villosa*. Different colour represent the 11 different types of LecRKs, and the red arrow indicates the LecRK-V.


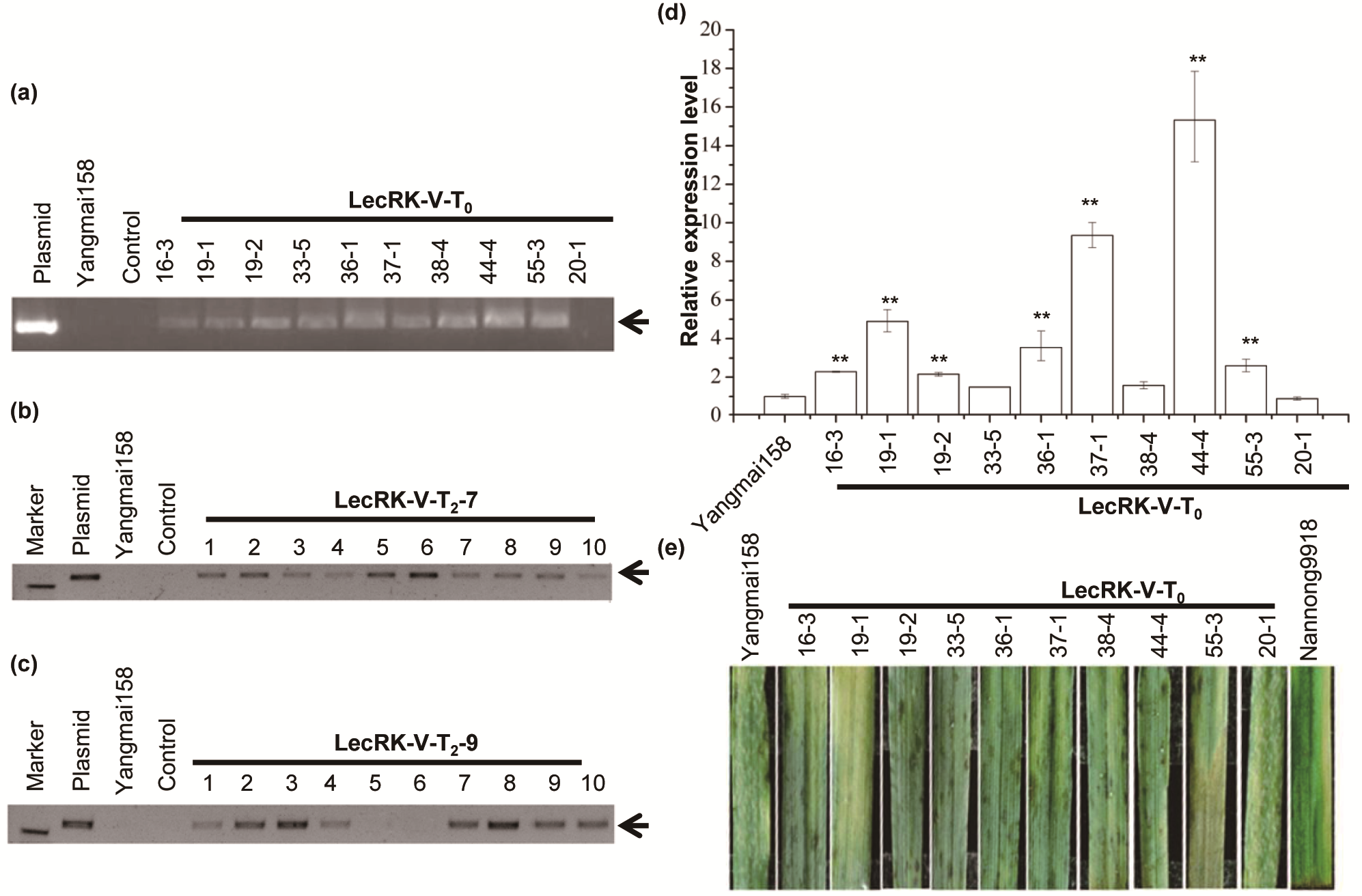


**Figure S2** Functional analysis of *LecRK-V* transgenic wheat. (a) In T_0_ generation, 9 positive *LecRK-V* transgenic plants, LecRK-V-T_0_-16-3, LecRK-V-T_0_-19-1, LecRK-V-T_0_-19-2, LecRK-V-T_0_-33-5, LecRK-V-T_0_-36-1, LecRK-V-T_0_-37-1, LecRK-V-T_0_-38-4, LecRK-V-T_0_-44-4 and LecRK-V-T_0_-55-3 were identified using PCR, and LecRK-V-T_0_-20-1 was negative transgenic plant. The arrow indicated the 624bp amplicon; (b-c) PCR identification of two positive transgenic lines at T_2_ generation, LecRK-V-T_2_-7(b) and LecRK-V-T_2_-9(c). The arrow indicated the 624bp amplicon; (d) The *LecRK-V* expression level in the leaves of 9 T_0_ positive transgenic plants, Yangmai158 and the negative transgenic plant (LecRK-V-T_0_-20-1), The quantitative RT-PCR values were normalized to those for *Tubulin*, and are presented as fold changes relative to Yangmai158, **p<0.01. (e) Powdery mildew resistance evaluation of 9 T_0_ positive transgenic plants, Yangmai158, Nannong9918 and the negative transgenic plant (LecRK-V-T_0_-20-1) using detached leaves at 7 dai with *Bgt* mixture.


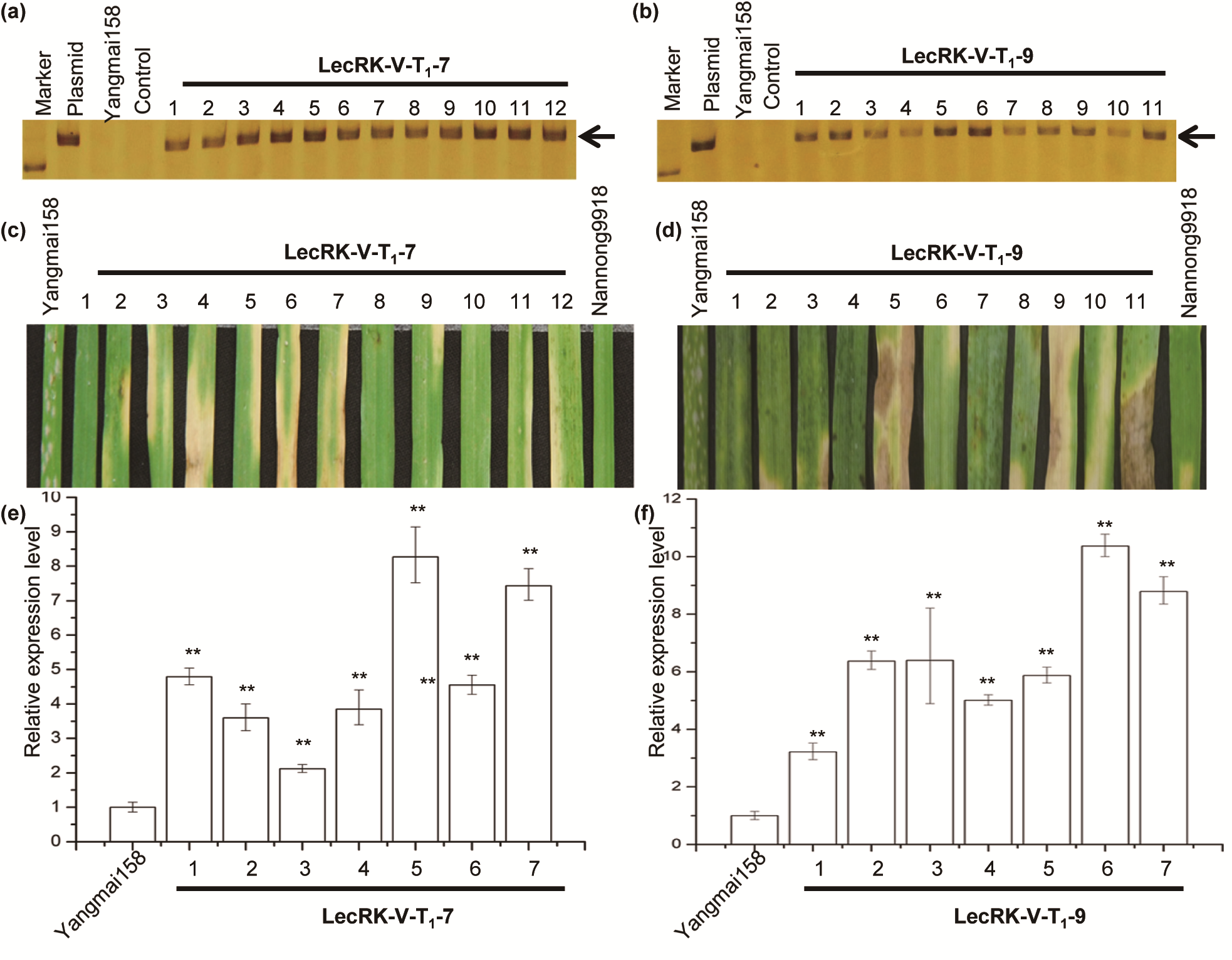


**Figure S3** Functional analysis of *LecRK-V* transgenic wheat at T_1_ generation. (a-b) PCR identification of LecRK-V-T_1_-7 (a) and LecRK-V-T_1_-9 (b). The arrows indicate the specific 624bp amplicon; (c-d) Powdery mildew resistance evaluation of LecRK-V-T_1_-7 (c) and LecRK-V-T_1_-9 (d) using detached leaves inoculated with *Bgt* mixture. Yangmai 158 and Nannong 9918 were used as susceptible and resistance controls. (e-f) The expression levels of *LecRK-V* in the leaves of LecRK-V-T_1_-7 (e) and LecRK-V-T_1_-9 (f), The quantitative RT-PCR values were normalized to those for *Tubulin*, and are presented as fold changes relative to Yangmai158, **p<0.01.

**
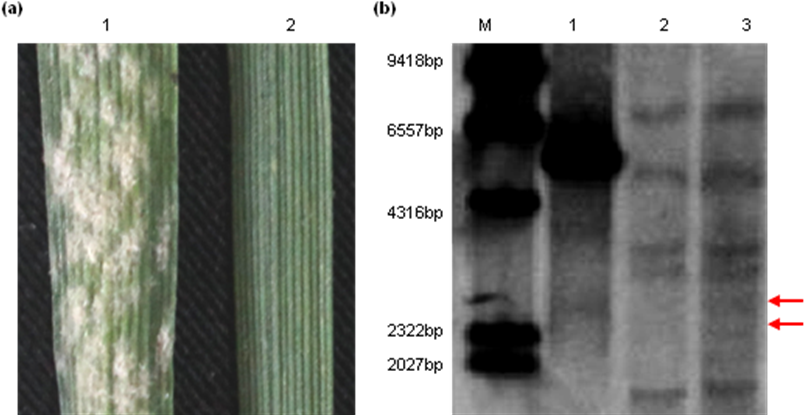
**

**Figure S4** Powdery mildew resistance evaluation and Southern blot of transgenic line LecRK-V-T_3_–2. (a) Powdery mildew resistance of LecRK-V-T_3_-2 using detached leaves inoculated with *Bgt* mixture. 1: Yangmai158; 2: LecRK-V-T_3_-2; (b) Southern blot of LecRK-V-T_3_–2. M: DNA marker; 1: plasmid *pBI*-220-*LecRK-V*; 2: Yangmai158; 3: LecRK-V-T_3_-2. *pBI*-220-*LecRK-V* plasmid and Yangmai158 were used as positive and negative controls, respectively. Red arrows indicate additional *LecRK-V* bands.


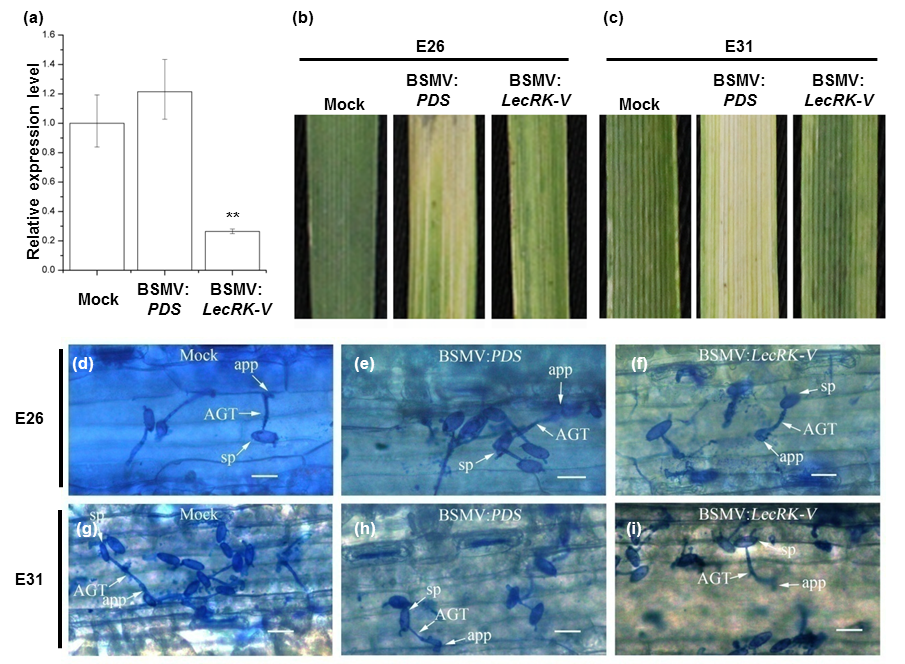


**Figure S5** VIGS of *LecRK-V* in *T. durum*-*H. villosa* amphiploid. (a) The VIGS efficacy showed by *LecRK-V* expression using qRT-PCR, The qRT-PCR values were normalized to those for *Tubulin*, and are presented as fold changes relative to Mock, **p<0.01; (b-c) The response of the fourth leaves of mock-inoculated, and BSMV:*PDS* or BSMV:*LecRK-V* inoculated plants to the infection of *Bgt* isolates E26 (b) or E31 (c); (d-f) The fungus development of E26 in mock-inoculated (d) and BSMV:*PDS* (e) or BSMV:*LecRK-V* inoculated plants (f); (g-i) The fungus development of E31 in mock-inoculated (g) and BSMV:*PDS* (h) or BSMV:*LecRK-V* inoculated plants (i). sp: spore; AGT: appressorial germ tube; app: appressorium penetration peg. Scale bar=10µM.

Table S1 Information of the primer pairs used in this study

| Primer name | Primer sequence | Used for |
| --- | --- | --- |
| LecRK-D-F | AYSTYBTCGCGTGGANYT | Homologous cloning |
| LecRK-D-R | ACKYTYCCRAAWSCMCC |
| LecRK-V-FL-F | ATGGCCTTGGTCGTGTGCC | Isolation of full-length *LecRK-V* |
| LecRK-V-FL-R | TCATCTTCCACCTGAGATG |
| LecRK-V-ChL-F | CTACCTGGCCCTCCTCAAC | Chromosome allocation |
| LecRK-V-ChL-R | AAATCCTTTGGTGGCATGG |
| LecRK-V-CL-F | CGGGATCCATGGCCTTGGTCGTGTGCCC | Sub-cellular localization vector construction |
| LecRK-V-CL-R | CGGCTAGCTCATCTTCCACCTGAGATGT |
| VIGS-LecRK-V-F | CGGCTAGCGCATCTACTTGGCGCCGGGG | VIGS vector construction |
| VIGS-LecRK-V-R | CGGCTAGCAGAGGTACTTATCGAGGCTG |
| CAMV35S-F | AGTTCATTTCATTTGGAGAGAACAC | Identification for transgenic plants |
| LecRK-V-SP-R | GCTGTGGCTATGGGCAGAACG |
| LecRK-V-Q-F | ACGCTTAGGGGACTTCG | qRT-PCR for *LecRKs* |
| LecRK-V-Q-R | CCTTCGCCCACAGGTTA |
| Tubulin-F | AGAACACTGTTGTAAGGCTCAAC | qRT-PCR for *Tubulin* |
| Tubulin-R | GAGCTTTACTGCCTCGAACATGG |
| TaNOX-Q-F | ATGCTCCAGTCCCTCAACCAT | qRT-PCR for *TaNOX* (AY561153.1) |
| TaNOX-Q-R | TTCTCCTTGTGGAACTCGAATTT |
| TaCAT-Q-F | TGCCTGTGTTTTTTATCCGAGA | qRT-PCR for *TaCAT* (HM989895.1) |
| TaCAT-Q-R | CTGCTGATTAAGGTGTAGGTGTT |
| TaAPX-Q-F | GGTTTGAGTGACCAGGACATTG | qRT-PCR for *TaAPX* (EF555121.1) |
| TaAPX-Q-R | GCATCCTCATCCGCAGCAT |
| TaGST-Q-F | GGAGCACAAGAGCCCCGAGC | qRT-PCR for *TaGST* (AJ441055) |
| TaGST-Q-R | CGGGTTGTAGGTGTGCGCGT |
| TaPR1-Q-F | CTGGAGCACGAAGCTGCAG | qRT-PCR for *TaPR1*(AF384143) |
| TaPR1-Q-R | CGAGTGCTGGAGCTTGCAGT |
| TaPR2-Q-F | GCAGCTCTACAGGTCCAAGG | qRT-PCR for *TaPR2*(DQ090946) |
| TaPR2-Q-R | CGGCGATGTACTTGATGTTG |

Table S2 The 275 LecRKs identified in wheat, barley, *T. urautu* and *Ae. tauschii*

| Name | Location | Amino acids | Clade in Fig S1 | Clade in Fig2 | Subclade in Fig2 |
| --- | --- | --- | --- | --- | --- |
| Hv-F2DTT9 | Chromosome 5: 518,526,056-518,528,197 | 571 | Ⅺ | Ⅰ | Ⅱ |
| Hv-F2DU83 | Chromosome 5: 506,158,292-506,161,244 | 684 | Ⅸ | Ⅰ | Ⅳ |
| Hv-F2E5K1 | [Chromosome 5: 528,173,840-528,176,197](http://plants.ensembl.org/Hordeum_vulgare/Location/View?db=core;g=MLOC_65972;r=5:528173840-528176197;t=MLOC_65972.1) | 674 | Ⅹ | Ⅰ | Ⅸ |
| Hv-F2E916 | Chromosome 5: 517,782,509-517,784,826 | 549 | Ⅹ | Ⅰ | Ⅵ |
| Hv-M0Y5U9 | [Chromosome 5: 528,178,597-528,181,209](http://plants.ensembl.org/Hordeum_vulgare/Location/View?db=core;g=MLOC_65973;r=5:528178597-528181209;t=MLOC_65973.1) | 538 | Ⅺ | Ⅰ | Ⅰ |
| Ta-W5CSA1 | Scaffold IWGSC_CSS_3B_scaff_10446081: 116-2,474 | 670 | Ⅹ | Ⅰ | Ⅵ |
| Ta-W5CUV3 | Scaffold IWGSC_CSS_3B_scaff_10686508: 9,692-11,584 | 491 | Ⅲ | Ⅱ | Ⅱ |
| Ta-W5CW51 | Scaffold IWGSC_CSS_3B_scaff_4115359: 2,539-4,551 | 670 | Ⅹ | Ⅰ | Ⅷ |
| Ta-W5CZG6 | Scaffold IWGSC_CSS_3B_scaff_10437849: 316-2,653 | 672 | Ⅹ | Ⅰ | Ⅴ |
| Ta-W5D6F4 | Scaffold IWGSC_CSS_3B_scaff_10761538: 2,403-4,693 | 671 | Ⅺ | Ⅰ | Ⅱ |
| Ta-W5D6W5 | Scaffold IWGSC_CSS_3B_scaff_8084914: 1,008-7,297 | 664 | Ⅰ | Ⅱ | Ⅰ |
| Ta-W5EZ92 | Scaffold IWGSC_CSS_5AL_scaff_2802452: 445-3,357 | 673 | Ⅹ | Ⅰ | Ⅸ |
| Ta-W5F2E0 | Scaffold IWGSC_CSS_5AL_scaff_2685050: 2,757-5,439 | 682 | Ⅺ | Ⅰ | Ⅰ |
| Ta-W5FDQ8 | Scaffold IWGSC_CSS_5BL_scaff_10827923: 8,423-10,984 | 681 | Ⅸ | Ⅰ | Ⅳ |
| Ta-W5FDT1 | Scaffold IWGSC_CSS_5BL_scaff_10849383: 9,367-11,681 | 488 | Ⅺ | Ⅰ | Ⅲ |
| Ta-W5FHV7 | Scaffold IWGSC_CSS_5BL_scaff_10891633: 5,074-7,999 | 629 | Ⅱ | Ⅱ | Ⅲ |
| Ta-W5FJ13 | Scaffold IWGSC_CSS_5BL_scaff_10893313: 1,262-6,273 | 708 | Ⅰ | Ⅱ | Ⅰ |
| Ta-W5FJU1 | Scaffold IWGSC_CSS_5BL_scaff_10849383: 12,231-14,925 | 703 | Ⅹ | Ⅰ | Ⅶ |
| Ta-W5FZ84 | Scaffold IWGSC_CSS_5DL_scaff_4606156: 4,641-7,179 | 715 | Ⅺ | Ⅰ | Ⅲ |
| Ta-W5G033 | Scaffold IWGSC_CSS_5DL_scaff_4574522: 1,872-5,357 | 707 | Ⅲ | Ⅱ | Ⅳ |
| Ta-W5G0P4 | Scaffold IWGSC_CSS_5DL_scaff_4556752: 1,764-3,718 | 426 | Ⅹ | Ⅰ | Ⅸ |
| Tu-M7ZL04 | SuperContig scaffold2223: 16,807-19,699 | 675 | Ⅱ | Ⅱ | Ⅲ |
| Tu-M7ZG89 | SuperContig scaffold21252: 33,675-38,818 | 681 | Ⅲ | Ⅱ | Ⅳ |
| Tu-M7ZXG6 | SuperContig scaffold25898: 35,535-37,541 | 668 | Ⅺ | Ⅰ | Ⅸ |
| Aet-M8CFD4 | SuperContig Scaffold24335: 145,693-147,714 | 673 | Ⅹ | Ⅰ | Ⅸ |
| Aet-M8C4C2 | SuperContig Scaffold24335: 151,180-153,204 | 674 | Ⅺ | Ⅰ | Ⅰ |
| Aet-M8BAK0 | SuperContig Scaffold24335: 125,655-128,805 | 611 | Ⅺ | Ⅰ | Ⅰ |
| Aet-M8C2H8 | SuperContig Scaffold64414: 12,339-14,354 | 671 | Ⅺ | Ⅰ | Ⅰ |
| Aet-M8AZG0 | SuperContig Scaffold76203: 39,006-41,404 | 673 | Ⅸ | Ⅰ | Ⅳ |
| Aet-M8C506 | SuperContig Scaffold60652: 10,633-12,780 | 715 | Ⅺ | Ⅰ | Ⅲ |
| Aet-M8B0E3 | SuperContig Scaffold60652: 7,860-10,082 | 670 | Ⅹ | Ⅰ | Ⅶ |
| Aet-M8BUU6 | SuperContig Scaffold78917: 61,154-64,621 | 734 | Ⅲ | Ⅱ | Ⅳ |
| Hv-F2CR98 | [Chromosome 6: 442,787,086-442,789,782](http://plants.ensembl.org/Hordeum_vulgare/Location/View?db=core;g=MLOC_70011;r=6:442787086-442789782) | 740 | Ⅰ | na | na |
| Hv-F2CUD7 | [Chromosome 7: 584,092,748-584,094,743](http://plants.ensembl.org/Hordeum_vulgare/Location/View?db=core;g=MLOC_73465;r=7:584092748-584094743;t=MLOC_73465.1) | 731 | Ⅱ | na | na |
| Hv-F2CY11 | Chromosome 2: 551,961,062-551,964,350 | 676 | Ⅷ | na | na |
| Hv-F2CYR3 | na | 483 | Ⅷ | na | na |
| Hv-F2CZB0 | [Chromosome 6:279168365-279170748](http://plants.ensembl.org/hordeum_vulgare/Location/View?db=core;g=MLOC_7248;r=6:279168365-279170748;t=MLOC_7248.1) | 675 | Ⅹ | na | na |
| Hv-F2D0V6 | na | 723 | Ⅱ | na | na |
| Hv-F2D4Y4 | [Chromosome 7: 558,839,964-558,842,364](http://plants.ensembl.org/Hordeum_vulgare/Location/View?db=core;g=MLOC_66502;r=7:558839964-558842364;t=MLOC_66502.1) | 694 | Ⅱ | na | na |
| Hv-F2D5A6 | na | 692 | Ⅷ | na | na |
| Hv-F2D696 | na | 692 | Ⅷ | na | na |
| Hv-F2D7N9 | [Chromosome 7: 91,285,478-91,288,278](http://plants.ensembl.org/Hordeum_vulgare/Location/View?db=core;g=MLOC_25651;r=7:91285478-91288278) | 681 | Ⅲ | na | na |
| Hv-F2D8T0 | [Chromosome 6:121297124-121299679](http://plants.ensembl.org/hordeum_vulgare/Location/View?db=core;g=MLOC_16150;r=6:121297124-121299679;t=MLOC_16150.1) | 684 | Ⅺ | na | na |
| Hv-F2D9V0 | [Chromosome 3: 41,527,630-41,530,277](http://plants.ensembl.org/Hordeum_vulgare/Location/View?db=core;g=MLOC_38752;r=3:41527630-41530277;t=MLOC_38752.1) | 673 | Ⅺ | na | na |
| Hv-F2DB91 | na | 692 | Ⅷ | na | na |
| Hv-F2DC87 | na | 690 | Ⅱ | na | na |
| Hv-F2DD36 | [Chromosome 7: 584,065,780-584,068,538](http://plants.ensembl.org/Hordeum_vulgare/Location/View?db=core;g=MLOC_64164;r=7:584065780-584068538;t=MLOC_64164.1) | 698 | Ⅱ | na | na |
| Hv-F2DF00 | [Chromosome 7: 2,597,463-2,599,767](http://plants.ensembl.org/Hordeum_vulgare/Location/View?db=core;g=MLOC_11202;r=7:2597463-2599767;t=MLOC_11202.1) | 684 | Ⅹ | na | na |
| Hv-F2DM09 | [Chromosome 5: 360,848,367-360,851,515](http://plants.ensembl.org/Hordeum_vulgare/Location/View?db=core;g=MLOC_76583;r=5:360848367-360851515;t=MLOC_76583.1) | 658 | Ⅳ | na | na |
| Hv-F2DNF5 | [Chromosome 4: 503,090,159-503,092,974](http://plants.ensembl.org/Hordeum_vulgare/Location/View?db=core;g=MLOC_66858;r=4:503090159-503092974;t=MLOC_66858.1) | 670 | Ⅵ | na | na |
| Hv-F2DR51 | [Chromosome 7: 583,868,151-583,871,062](http://plants.ensembl.org/Hordeum_vulgare/Location/View?db=core;g=MLOC_61829;r=7:583868151-583871062;t=MLOC_61829.1) | 658 | Ⅱ | na | na |
| Hv-F2DRE6 | [Chromosome 7: 583,868,151-583,871,062](http://plants.ensembl.org/Hordeum_vulgare/Location/View?db=core;g=MLOC_61829;r=7:583868151-583871062;t=MLOC_61829.1) | 658 | Ⅱ | na | na |
| Hv-F2DXG0 | Chromosome 1: 60,325,969-60,327,077 | 741 | Ⅳ | na | na |
| Hv-F2E108 | [Chromosome 7HS: 493,884-496,607](http://plants.ensembl.org/Hordeum_vulgare/Location/View?db=core;g=MLOC_5877;r=7HS:493884-496607;t=MLOC_5877.1) | 733 | Ⅰ | na | na |
| Hv-F2E1C0 | [Chromosome 2: 623,386,310-623,388,706](http://plants.ensembl.org/Hordeum_vulgare/Location/View?db=core;g=MLOC_56716;r=2:623386310-623388706) | 730 | Ⅲ | na | na |
| Hv-F2E1Y7 | [Chromosome 1: 462,965,137-462,971,156](http://plants.ensembl.org/Hordeum_vulgare/Location/View?db=core;g=MLOC_15040;r=1:462965137-462971156) | 721 | Ⅲ | na | na |
| Hv-F2E428 | [Chromosome 3: 463,919,860-463,922,285](http://plants.ensembl.org/Hordeum_vulgare/Location/View?db=core;g=MLOC_60699;r=3:463919860-463922285;t=MLOC_60699.1) | 671 | Ⅲ | na | na |
| Hv-F2E5Z5 | [Chromosome 7: 28,136,928-28,138,412](http://plants.ensembl.org/Hordeum_vulgare/Location/View?db=core;g=MLOC_68841;r=7:28136928-28138412;t=MLOC_68841.1) | 673 | Ⅶ | na | na |
| Hv-F2E6D0 | Chromosome 5: 3,133,763-3,137,865 | 716 | Ⅲ | na | na |
| Hv-F2E7I8 | Chromosome 7: 54,486,931-54,491,157 | 688 | Ⅺ | na | na |
| Hv-F2E9Q4 | na | 500 | Ⅴ | na | na |
| Hv-F2E9W1 | [Chromosome 5: 517,782,509-517,784,826](http://plants.ensembl.org/Hordeum_vulgare/Location/View?db=core;g=MLOC_5810;r=5:517782509-517784826;t=MLOC_5810.1) | 389 | Ⅹ | na | na |
| Hv-F2EHC4 | Chromosome 4: 134,391,978-134,394,830 | 720 | Ⅲ | na | na |
| Hv-F2EIG3 | [Chromosome 2: 579,982,483-579,985,039](http://plants.ensembl.org/Hordeum_vulgare/Location/View?db=core;g=MLOC_66280;r=2:579982483-579985039;t=MLOC_66280.1) | 699 | Ⅰ | na | na |
| Hv-M0UIA5 | [Chromosome 7: 2,597,463-2,599,767](http://plants.ensembl.org/Hordeum_vulgare/Location/View?db=core;g=MLOC_11202;r=7:2597463-2599767;t=MLOC_11202.1) | 686 | Ⅹ | na | na |
| Hv-M0UWL7 | [Chromosome 1: 462,965,137-462,971,156](http://plants.ensembl.org/Hordeum_vulgare/Location/View?db=core;g=MLOC_15040;r=1:462965137-462971156) | 749 | Ⅲ | na | na |
| Hv-M0UWL8 | [Chromosome 1: 462,965,137-462,971,156](http://plants.ensembl.org/Hordeum_vulgare/Location/View?db=core;g=MLOC_15040;r=1:462965137-462971156) | 470 | Ⅲ | na | na |
| Hv-M0UWL9 | [Chromosome 1: 462,965,137-462,971,156](http://plants.ensembl.org/Hordeum_vulgare/Location/View?db=core;g=MLOC_15040;r=1:462965137-462971156) | 559 | Ⅲ | na | na |
| Hv-M0UWM0 | [Chromosome 1: 462,965,137-462,971,156](http://plants.ensembl.org/Hordeum_vulgare/Location/View?db=core;g=MLOC_15040;r=1:462965137-462971156) | 517 | Ⅲ | na | na |
| Hv-M0UWY8 | [Chromosome 2: 9,865,779-9,868,280](http://plants.ensembl.org/Hordeum_vulgare/Location/View?db=core;g=MLOC_15183;r=2:9865779-9868280;t=MLOC_15183.1) | 652 | Ⅴ | na | na |
| Hv-M0VB12 | Chromosome 6: 365,114,100-365,116,454 | 670 | Ⅷ | na | na |
| Hv-M0VF36 | Chromosome 7: 91,285,478-91,288,278 | 703 | Ⅲ | na | na |
| Hv-M0VHH7 | Chromosome 7: 340,793,766-340,796,970 | 757 | Ⅰ | na | na |
| Hv-M0VR30 | [Chromosome 2: 551,961,062-551,964,350](http://plants.ensembl.org/Hordeum_vulgare/Location/View?db=core;g=MLOC_36562;r=2:551961062-551964350) | 676 | Ⅷ | na | na |
| Hv-M0VR31 | [Chromosome 2: 551,961,062-551,964,350](http://plants.ensembl.org/Hordeum_vulgare/Location/View?db=core;g=MLOC_36562;r=2:551961062-551964350) | 637 | Ⅷ | na | na |
| Hv-M0VUE6 | Chromosome 7: 27,654,703-27,656,188 | 452 | Ⅷ | na | na |
| Hv-M0VVC5 | [Chromosome 7: 54,486,931-54,491,157](http://plants.ensembl.org/Hordeum_vulgare/Location/View?db=core;g=MLOC_37576;r=7:54486931-54491157) | 688 | Ⅺ | na | na |
| Hv-M0VXQ0 | [Chromosome 5: 518,526,056-518,528,197](http://plants.ensembl.org/Hordeum_vulgare/Location/View?db=core;g=MLOC_38262;r=5:518526056-518528197;t=MLOC_38262.1) | 671 | Ⅺ | na | na |
| Hv-M0W0N4 | [Chromosome 2: 27,549,727-27,552,164](http://plants.ensembl.org/Hordeum_vulgare/Location/View?db=core;g=MLOC_39533;r=2:27549727-27552164;t=MLOC_39533.1) | 686 | Ⅷ | na | na |
| Hv-M0W8J7 | [Chromosome 3: 41,511,430-41,513,828](http://plants.ensembl.org/Hordeum_vulgare/Location/View?db=core;g=MLOC_4492;r=3:41511430-41513828;t=MLOC_4492.1) | 673 | Ⅹ | na | na |
| Hv-M0WQ14 | [Chromosome 1: 231,569,056-231,571,500](http://plants.ensembl.org/Hordeum_vulgare/Location/View?db=core;g=MLOC_5365;r=1:231569056-231571500;t=MLOC_5365.1) | 674 | Ⅵ | na | na |
| Hv-M0WRJ6 | [Chromosome 3: 140,031,123-140,038,633](http://plants.ensembl.org/Hordeum_vulgare/Location/View?db=core;g=MLOC_54049;r=3:140031123-140038633;t=MLOC_54049.1) | 686 | Ⅰ | na | na |
| Hv-M0X174 | [Chromosome 5: 3,133,763-3,137,865](http://plants.ensembl.org/Hordeum_vulgare/Location/View?db=core;g=MLOC_56180;r=5:3133763-3137865) | 622 | Ⅲ | na | na |
| Hv-M0X175 | [Chromosome 5: 3,133,763-3,137,865](http://plants.ensembl.org/Hordeum_vulgare/Location/View?db=core;g=MLOC_56180;r=5:3133763-3137865) | 715 | Ⅲ | na | na |
| Hv-M0X3H1 | Chromosome 2: 623,386,310-623,388,706 | 488 | Ⅲ | na | na |
| Hv-M0X8J3 | [Chromosome 4: 134,391,978-134,394,830](http://plants.ensembl.org/Hordeum_vulgare/Location/View?db=core;g=MLOC_57881;r=4:134391978-134394830) | 720 | Ⅲ | na | na |
| Hv-M0X8J4 | [Chromosome 4: 134,391,978-134,394,830](http://plants.ensembl.org/Hordeum_vulgare/Location/View?db=core;g=MLOC_57881;r=4:134391978-134394830) | 471 | Ⅲ | na | na |
| Hv-M0X9E8 | [Chromosome 5: 517,782,509-517,784,826](http://plants.ensembl.org/Hordeum_vulgare/Location/View?db=core;g=MLOC_5810;r=5:517782509-517784826;t=MLOC_5810.1) | 595 | Ⅹ | na | na |
| Hv-M0X9E9 | Chromosome 1: 61,930,071-61,933,350 | 665 | Ⅵ | na | na |
| Hv-M0X9F1 | Chromosome 1: 61,930,071-61,933,350 | 630 | Ⅵ | na | na |
| Hv-M0XC88 | [Chromosome 7HS: 493,884-496,607](http://plants.ensembl.org/Hordeum_vulgare/Location/View?db=core;g=MLOC_5877;r=7HS:493884-496607;t=MLOC_5877.1) | 573 | Ⅺ | na | na |
| Hv-M0XCY1 | Chromosome 7: 9,300,213-9,303,667 | 479 | Ⅱ | na | na |
| Hv-M0XCY2 | Chromosome 7: 9,300,213-9,303,667 | 696 | Ⅱ | na | na |
| Hv-M0XJV7 | Chromosome 3: 463,919,860-463,922,285 | 672 | Ⅲ | na | na |
| Hv-M0XP82 | [Chromosome 7: 583,868,151-583,871,062](http://plants.ensembl.org/Hordeum_vulgare/Location/View?db=core;g=MLOC_61829;r=7:583868151-583871062;t=MLOC_61829.1) | 650 | Ⅱ | na | na |
| Hv-M0XPY3 | [Chromosome 7: 492,801-494,899](http://plants.ensembl.org/Hordeum_vulgare/Location/View?db=core;g=MLOC_62002;r=7:492801-494899;t=MLOC_62002.1) | 691 | Ⅺ | na | na |
| Hv-M0XX38 | [Chromosome 2: 27,987,937-27,990,436](http://plants.ensembl.org/Hordeum_vulgare/Location/View?db=core;g=MLOC_63818;r=2:27987937-27990436;t=MLOC_63818.1) | 689 | Ⅷ | na | na |
| Hv-M0XY87 | [Chromosome 7: 584,065,780-584,068,538](http://plants.ensembl.org/Hordeum_vulgare/Location/View?db=core;g=MLOC_64164;r=7:584065780-584068538;t=MLOC_64164.1) | 698 | Ⅱ | na | na |
| Hv-M0Y5U8 | [Chromosome 5: 528,173,840-528,176,197](http://plants.ensembl.org/Hordeum_vulgare/Location/View?db=core;g=MLOC_65972;r=5:528173840-528176197;t=MLOC_65972.1) | 674 | Ⅹ | na | na |
| Hv-M0Y7L1 | [Chromosome 7: 558,839,964-558,842,364](http://plants.ensembl.org/Hordeum_vulgare/Location/View?db=core;g=MLOC_66502;r=7:558839964-558842364;t=MLOC_66502.1) | 694 | Ⅱ | na | na |
| Hv-M0Y844 | [Chromosome 5: 506,158,292-506,161,244](http://plants.ensembl.org/Hordeum_vulgare/Location/View?db=core;g=MLOC_66630;r=5:506158292-506161244;t=MLOC_66630.1) | 684 | Ⅸ | na | na |
| Hv-M0Y8Y9 | [Chromosome 4: 503,090,159-503,092,974](http://plants.ensembl.org/Hordeum_vulgare/Location/View?db=core;g=MLOC_66858;r=4:503090159-503092974;t=MLOC_66858.1) | 670 | Ⅵ | na | na |
| Hv-M0YDH8 | Chromosome 6: 273,470,015-273,472,178 | 666 | Ⅺ | na | na |
| Hv-M0YFM7 | [Chromosome 7: 28,136,928-28,138,412](http://plants.ensembl.org/Hordeum_vulgare/Location/View?db=core;g=MLOC_68841;r=7:28136928-28138412;t=MLOC_68841.1) | 444 | Ⅶ | na | na |
| Hv-M0YJP2 | [Chromosome 6: 442,787,086-442,789,782](http://plants.ensembl.org/Hordeum_vulgare/Location/View?db=core;g=MLOC_70011;r=6:442787086-442789782) | 610 | Ⅰ | na | na |
| Hv-M0YK33 | [Chromosome 7: 291,083,056-291,087,985](http://plants.ensembl.org/Hordeum_vulgare/Location/View?db=core;g=MLOC_7012;r=7:291083056-291087985) | 497 | Ⅲ | na | na |
| Hv-M0YQC3 | [Chromosome 1: 394,984,020-394,987,628](http://plants.ensembl.org/Hordeum_vulgare/Location/View?db=core;g=MLOC_71685;r=1:394984020-394987628) | 544 | Ⅳ | na | na |
| Hv-M0YQC4 | [Chromosome 1: 394,984,020-394,987,628](http://plants.ensembl.org/Hordeum_vulgare/Location/View?db=core;g=MLOC_71685;r=1:394984020-394987628) | 545 | Ⅳ | na | na |
| Hv-M0YVI8 | [Chromosome 7: 584,092,748-584,094,743](http://plants.ensembl.org/Hordeum_vulgare/Location/View?db=core;g=MLOC_73465;r=7:584092748-584094743;t=MLOC_73465.1) | 596 | Ⅱ | na | na |
| Hv-M0YXH9 | Chromosome 2: 611,249,684-611,252,009 | 650 | Ⅺ | na | na |
| Hv-M0Z5W4 | [Chromosome 7: 584,604,242-584,606,048](http://plants.ensembl.org/Hordeum_vulgare/Location/View?db=core;g=MLOC_7765;r=7:584604242-584606048;t=MLOC_7765.1) | 592 | Ⅱ | na | na |
| Hv-M0Z6K5 | Chromosome 2: 575,512,326-575,514,706 | 720 | Ⅰ | na | na |
| Hv-M0Z6K6 | Chromosome 2: 575,512,326-575,514,706 | 586 | Ⅰ | na | na |
| Hv-Q6A1H8 | na | 692 | Ⅷ | na | na |
| Ta-A0A077RAP2 | na | 533 | Ⅱ | na | na |
| Ta-A0A077RP04 | na | 589 | Ⅱ | na | na |
| Ta-A0A077RPT3 | na | 592 | Ⅱ | na | na |
| Ta-A0A077RS98 | na | 659 | Ⅱ | na | na |
| Ta-A0A077RUQ5 | na | 680 | Ⅺ | na | na |
| Ta-A0A077RV77 | na | 658 | Ⅱ | na | na |
| Ta-A0A077RVH7 | na | 626 | Ⅰ | na | na |
| Ta-A0A077RVN0 | na | 688 | Ⅱ | na | na |
| Ta-A0A077RVW7 | na | 675 | Ⅲ | na | na |
| Ta-A0A077RZ12 | na | 620 | Ⅺ | na | na |
| Ta-A0A077S6Z4 | na | 1341 | Ⅺ | na | na |
| Ta-A0A077S7I3 | na | 696 | Ⅰ | na | na |
| Ta-B8XSM8 | Scaffold IWGSC_CSS_4AL_scaff_7077302: 8,934-11,547 | 673 | Ⅷ | na | na |
| Ta-B8XSM9 | Scaffold IWGSC_CSS_4AL_scaff_7077302: 8,934-11,547 | 676 | Ⅶ | na | na |
| Ta-B8XSN3 | Scaffold IWGSC_CSS_4AL_scaff_7077302: 8,934-11,547 | 671 | Ⅷ | na | na |
| Ta-E7CWB1 | Scaffold IWGSC_CSS_4AL_scaff_7077302: 8,934-11,547 | 673 | Ⅷ | na | na |
| Ta-E7CWB5 | Scaffold IWGSC_CSS_4AL_scaff_7077302: 8,934-11,547 | 666 | Ⅷ | na | na |
| Ta-W4ZXF8 | Scaffold IWGSC_CSS_1AS_scaff_3313964: 2,118-4,289 | 669 | Ⅹ | na | na |
| Ta-W5AAP9 | Scaffold IWGSC_CSS_1BS_scaff_3423601: 21,773-24,822 | 667 | Ⅺ | na | na |
| Ta-W5AM06 | Scaffold IWGSC_CSS_1DS_scaff_1876216: 4-2,596 | 740 | Ⅳ | na | na |
| Ta-W5AMW1 | Scaffold IWGSC_CSS_1DS_scaff_1915947: 6,172-8,922 | 667 | Ⅺ | na | na |
| Ta-W5AND9 | Scaffold IWGSC_CSS_1DS_scaff_1915947: 2,520-4,908 | 668 | Ⅹ | na | na |
| Ta-W5AZ54 | Scaffold IWGSC_CSS_2AS_scaff_5240395: 2,871-4,952 | 693 | Ⅲ | na | na |
| Ta-W5AZZ8 | Scaffold IWGSC_CSS_2AS_scaff_5302288: 5,334-7,337 | 667 | Ⅺ | na | na |
| Ta-W5B4R4 | Scaffold IWGSC_CSS_2AS_scaff_5208440: 9,775-12,037 | 672 | Ⅹ | na | na |
| Ta-W5B572 | Scaffold IWGSC_CSS_2AS_scaff_5243523: 7,629-9,413 | 594 | Ⅶ | na | na |
| Ta-W5B686 | Scaffold IWGSC_CSS_2BL_scaff_2246582: 1,413-3,474 | 517 | Ⅰ | na | na |
| Ta-W5B7Q1 | Scaffold IWGSC_CSS_2BL_scaff_3694842: 1-2,050 | 577 | Ⅰ | na | na |
| Ta-W5B8X0 | Scaffold IWGSC_CSS_2BL_scaff_8044964: 3,321-5,755 | 576 | Ⅲ | na | na |
| Ta-W5BIG0 | Scaffold IWGSC_CSS_2BS_scaff_5246248: 412-6,465 | 675 | Ⅹ | na | na |
| Ta-W5BL53 | Scaffold IWGSC_CSS_2BS_scaff_5191341: 13,665-16,252 | 679 | Ⅶ | na | na |
| Ta-W5BLI4 | Scaffold IWGSC_CSS_2BS_scaff_5161932: 2,863-5,152 | 670 | Ⅷ | na | na |
| Ta-W5BLZ3 | Scaffold IWGSC_CSS_2BS_scaff_5162705: 2,166-4,461 | 677 | Ⅶ | na | na |
| Ta-W5BML1 | Scaffold IWGSC_CSS_2BS_scaff_5199181: 13,475-15,736 | 630 | Ⅶ | na | na |
| Ta-W5BNP7 | Scaffold IWGSC_CSS_2BS_scaff_5247777: 12,186-14,267 | 693 | Ⅲ | na | na |
| Ta-W5BNQ0 | Scaffold IWGSC_CSS_2BS_scaff_5240221: 6,490-8,669 | 652 | Ⅴ | na | na |
| Ta-W5BPE1 | Scaffold IWGSC_CSS_2BS_scaff_5199181: 17,713-21,159 | 679 | Ⅷ | na | na |
| Ta-W5BSM0 | Scaffold IWGSC_CSS_2DL_scaff_9874690: 1,356-2,994 | 540 | Ⅲ | na | na |
| Ta-W5C404 | Scaffold IWGSC_CSS_2DS_scaff_5356359: 5,440-7,842 | 556 | Ⅶ | na | na |
| Ta-W5C594 | Scaffold IWGSC_CSS_2DS_scaff_5325753: 2,772-4,853 | 693 | Ⅲ | na | na |
| Ta-W5C8D0 | Scaffold IWGSC_CSS_2DS_scaff_5330846: 1,490-3,781 | 629 | Ⅴ | na | na |
| Ta-W5C9Q6 | Scaffold IWGSC_CSS_2DS_scaff_5377399: 4,664-6,718 | 684 | Ⅶ | na | na |
| Ta-W5CLE2 | Scaffold IWGSC_CSS_3AS_scaff_3441426: 2-2,123 | 597 | Ⅹ | na | na |
| Ta-W5DMW0 | Scaffold IWGSC_CSS_4AL_scaff_7150007: 3,766-6,422 | 713 | Ⅲ | na | na |
| Ta-W5EAF0 | Scaffold IWGSC_CSS_4BS_scaff_4923111: 3,800-6,201 | 710 | Ⅲ | na | na |
| Ta-W5EAY7 | Scaffold IWGSC_CSS_4BS_scaff_4868257: 2,710-4,872 | 721 | Ⅱ | na | na |
| Ta-W5ETD8 | Scaffold IWGSC_CSS_4DS_scaff_2310089: 8,197-11,104 | 713 | Ⅲ | na | na |
| Ta-W5FM00 | Scaffold IWGSC_CSS_5BS_scaff_974261: 2-1,994 | 583 | Ⅵ | na | na |
| Ta-W5FP28 | Scaffold IWGSC_CSS_5BS_scaff_2230444: 8,302-11,595 | 719 | Ⅱ | na | na |
| Ta-W5G3A8 | Scaffold IWGSC_CSS_5DS_scaff_2754894: 2,214-4,869 | 693 | Ⅵ | na | na |
| Ta-W5G465 | Scaffold IWGSC_CSS_5DS_scaff_2758801: 2,128-4,305 | 725 | Ⅺ | na | na |
| Ta-W5G5I8 | Scaffold IWGSC_CSS_6AL_scaff_5776112: 3-2,663 | 573 | Ⅺ | na | na |
| Ta-W5G644 | Scaffold IWGSC_CSS_6AL_scaff_5810066: 15,100-16,746 | 516 | Ⅹ | na | na |
| Ta-W5GAD8 | Scaffold IWGSC_CSS_6AL_scaff_5830594: 4,517-7,459 | 477 | Ⅹ | na | na |
| Ta-W5GD44 | Scaffold IWGSC_CSS_6AS_scaff_4352999: 15,012-18,710 | 704 | Ⅲ | na | na |
| Ta-W5GTY6 | Scaffold IWGSC_CSS_6DL_scaff_3231994: 9,144-10,793 | 517 | Ⅹ | na | na |
| Ta-W5GUL8 | Scaffold IWGSC_CSS_6DL_scaff_3257449: 8,139-10,435 | 667 | Ⅷ | na | na |
| Ta-W5GVT5 | Scaffold IWGSC_CSS_6DL_scaff_1272220: 211-2,387 | 501 | Ⅰ | na | na |
| Ta-W5GWA8 | Scaffold IWGSC_CSS_6DL_scaff_3299490: 775-9,803 | 671 | Ⅺ | na | na |
| Ta-W5GX67 | Scaffold IWGSC_CSS_6DL_scaff_3301284: 6,916-9,137 | 673 | Ⅹ | na | na |
| Ta-W5H3G6 | Scaffold IWGSC_CSS_6DS_scaff_2084218: 3-2,105 | 609 | Ⅺ | na | na |
| Ta-W5H597 | Scaffold IWGSC_CSS_6DS_scaff_2124633: 1,374-3,807 | 679 | Ⅹ | na | na |
| Ta-W5HGE2 | Scaffold IWGSC_CSS_7AS_scaff_4249604: 243-1,988 | 580 | Ⅹ | na | na |
| Ta-W5HKD6 | Scaffold IWGSC_CSS_7BL_scaff_6484330: 78-1,431 | 402 | Ⅲ | na | na |
| Ta-W5I3G7 | Scaffold IWGSC_CSS_7DL_scaff_3351478: 12,464-14,996 | 695 | Ⅱ | na | na |
| Tu-M7Y5H2 | SuperContig scaffold79752: 10,280-13,793 | 598 | Ⅷ | na | na |
| Tu-M7Y9L8 | SuperContig scaffold8136: 37,706-39,706 | 667 | Ⅺ | na | na |
| Tu-M7YC97 | SuperContig scaffold8024: 57,930-64,014 | 550 | Ⅲ | na | na |
| Tu-M7YF28 | SuperContig scaffold125040: 29,619-31,758 | 480 | Ⅺ | na | na |
| Tu-M7YFL8 | SuperContig scaffold70024: 20,252-22,379 | 664 | Ⅺ | na | na |
| Tu-M7YG67 | SuperContig scaffold125040: 25,729-27,774 | 681 | Ⅹ | na | na |
| Tu-M7YHU0 | SuperContig scaffold8136: 28,356-32,109 | 661 | Ⅹ | na | na |
| Tu-M7YKB0 | SuperContig scaffold205302: 21,006-23,572 | 642 | Ⅹ | na | na |
| Tu-M7YRF3 | SuperContig scaffold95694: 65,278-69,222 | 724 | Ⅱ | na | na |
| Tu-M7YT18 | SuperContig scaffold68693: 63,586-65,745 | 564 | Ⅱ | na | na |
| Tu-M7YU88 | SuperContig scaffold6053: 8,730-11,008 | 623 | Ⅷ | na | na |
| Tu-M7YUV0 | SuperContig scaffold31677: 118,223-121,136 | 572 | Ⅰ | na | na |
| Tu-M7YY54 | SuperContig scaffold64141: 68,851-72,940 | 541 | Ⅰ | na | na |
| Tu-M7Z0W1 | SuperContig scaffold31664: 58,444-60,447 | 667 | Ⅷ | na | na |
| Tu-M7Z2Q8 | SuperContig scaffold39880: 42,685-44,688 | 667 | Ⅺ | na | na |
| Tu-M7Z5S7 | SuperContig scaffold109: 100,379-102,358 | 659 | Ⅶ | na | na |
| Tu-M7Z7K8 | SuperContig scaffold55019: 43,916-45,925 | 669 | Ⅴ | na | na |
| Tu-M7Z873 | SuperContig scaffold27420: 35,201-36,979 | 592 | Ⅹ | na | na |
| Tu-M7ZGC1 | SuperContig scaffold34110: 57,071-59,091 | 653 | Ⅹ | na | na |
| Tu-M7ZGI3 | SuperContig scaffold55019: 40,820-43,229 | 699 | Ⅴ | na | na |
| Tu-M7ZS11 | SuperContig scaffold27442: 22,607-25,143 | 524 | Ⅰ | na | na |
| Tu-M7ZVI7 | SuperContig scaffold115014: 35,247-36,710 | 487 | Ⅹ | na | na |
| Tu-M7ZXQ6 | SuperContig scaffold18356: 36,080-38,668 | 635 | Ⅲ | na | na |
| Tu-M8A5Y7 | SuperContig scaffold10601: 51,194-75,122 | 690 | Ⅰ | na | na |
| Tu-M8A7E8 | SuperContig scaffold132401: 30,247-32,325 | 693 | Ⅲ | na | na |
| Tu-M8AD26 | SuperContig scaffold28394: 103,388-105,454 | 688 | Ⅱ | na | na |
| Tu-M8AIG6 | SuperContig scaffold43346: 56,987-58,429 | 480 | Ⅶ | na | na |
| Tu-M8AN24 | SuperContig scaffold2689: 83,499-91,860 | 409 | Ⅵ | na | na |
| Tu-M8ANZ8 | SuperContig scaffold28394: 75,330-77,237 | 635 | Ⅱ | na | na |
| Tu-M8AQC6 | SuperContig scaffold35233: 15,315-16,757 | 480 | Ⅶ | na | na |
| Tu-M8AZT4 | SuperContig scaffold1489: 71,631-73,966 | 746 | Ⅲ | na | na |
| Tu-T1LCI8 | SuperContig scaffold109: 110,361-113,102 | 685 | Ⅶ | na | na |
| Tu-T1LGN6 | SuperContig scaffold4006: 77,280-83,099 | 581 | Ⅵ | na | na |
| Tu-T1LP26 | SuperContig scaffold35233: 20,585-22,585 | 667 | Ⅷ | na | na |
| Tu-T1LQX3 | SuperContig scaffold59892: 40,863-42,737 | 625 | Ⅰ | na | na |
| Tu-T1MPB7 | SuperContig scaffold56105: 18,071-20,968 | 650 | Ⅱ | na | na |
| Tu-T1MZQ9 | SuperContig scaffold70043: 35,154-37,042 | 592 | Ⅵ | na | na |
| Tu-T1N593 | SuperContig scaffold72798: 45,807-64,588 | 1149 | Ⅵ | na | na |
| Tu-T1NM97 | SuperContig scaffold35771: 19,025-21,320 | 679 | Ⅱ | na | na |
| Tu-T1NPA3 | SuperContig scaffold29077: 64,708-66,771 | 688 | Ⅰ | na | na |
| Tu-T1NUI4 | SuperContig scaffold22145: 66,906-68,355 | 429 | Ⅳ | na | na |
| Aet-B8XSN6 | na | 667 | Ⅷ | na | na |
| Aet-B8XSN7 | na | 677 | Ⅶ | na | na |
| Aet-M8AM75 | SuperContig Scaffold86487: 9,012-13,666 | 651 | Ⅰ | na | na |
| Aet-M8AU55 | SuperContig Scaffold89969: 16,471-18,504 | 677 | Ⅲ | na | na |
| Aet-M8AWR5 | SuperContig Scaffold84712: 14,020-16,059 | 679 | Ⅶ | na | na |
| Aet-M8AYP4 | SuperContig Scaffold78445: 9,740-11,728 | 662 | Ⅴ | na | na |
| Aet-M8B1J2 | SuperContig Scaffold58756: 8,898-13,796 | 734 | Ⅱ | na | na |
| Aet-M8B3P6 | SuperContig Scaffold68279: 34,737-36,851 | 704 | Ⅰ | na | na |
| Aet-M8B4T0 | SuperContig Scaffold68279: 14,702-18,300 | 687 | Ⅰ | na | na |
| Aet-M8BEB2 | SuperContig Scaffold52792: 68,634-70,796 | 619 | Ⅱ | na | na |
| Aet-M8BJ64 | SuperContig Scaffold24521: 54,424-56,809 | 510 | Ⅴ | na | na |
| Aet-M8BLR0 | SuperContig Scaffold73009: 22,514-25,496 | 698 | Ⅱ | na | na |
| Aet-M8BMW9 | SuperContig Scaffold14289: 1,676-5,968 | 853 | Ⅲ | na | na |
| Aet-M8BN39 | SuperContig Scaffold70328: 22,746-24,434 | 443 | Ⅷ | na | na |
| Aet-M8BN54 | SuperContig Scaffold21123: 50,178-51,899 | 513 | Ⅳ | na | na |
| Aet-M8BRI0 | SuperContig Scaffold64414: 17,869-19,899 | 676 | Ⅹ | na | na |
| Aet-M8BRM9 | SuperContig Scaffold18487: 60,118-62,162 | 595 | Ⅶ | na | na |
| Aet-M8BW31 | SuperContig Scaffold14685: 5,873-7,872 | 595 | Ⅵ | na | na |
| Aet-M8BXZ8 | SuperContig Scaffold13194: 63,337-65,361 | 674 | Ⅺ | na | na |
| Aet-M8BYK0 | SuperContig Scaffold13194: 39,120-41,418 | 725 | Ⅹ | na | na |
| Aet-M8BYZ5 | SuperContig Scaffold70469: 13,908-16,195 | 706 | Ⅷ | na | na |
| Aet-M8BZM8 | SuperContig Scaffold12401: 88,412-90,519 | 669 | Ⅱ | na | na |
| Aet-M8C175 | SuperContig Scaffold5008: 49,293-54,751 | 1051 | Ⅶ | na | na |
| Aet-M8C3X5 | SuperContig Scaffold24535: 27,514-29,823 | 496 | Ⅺ | na | na |
| Aet-M8C9N4 | SuperContig Scaffold5456: 134,820-138,567 | 749 | Ⅵ | na | na |
| Aet-M8C9N8 | SuperContig Scaffold5008: 44,777-47,048 | 681 | Ⅶ | na | na |
| Aet-M8CBU6 | SuperContig Scaffold18163: 116,910-118,964 | 653 | Ⅱ | na | na |
| Aet-M8CCG4 | SuperContig Scaffold3205: 34,933-37,306 | 721 | Ⅲ | na | na |
| Aet-M8CEP6 | SuperContig Scaffold2273: 160,342-162,354 | 670 | Ⅹ | na | na |
| Aet-M8CM62 | SuperContig Scaffold9939: 20,608-22,705 | 643 | Ⅷ | na | na |
| Aet-M8CQT0 | SuperContig Scaffold14915: 13,389-17,215 | 569 | Ⅲ | na | na |
| Aet-M8CVM0 | SuperContig Scaffold3914: 6,856-8,910 | 684 | Ⅺ | na | na |
| Aet-M8CY76 | SuperContig Scaffold2273: 155,764-157,788 | 674 | Ⅺ | na | na |
| Aet-M8D0Q7 | SuperContig Scaffold7542: 13,579-15,651 | 690 | Ⅱ | na | na |
| Aet-M8D1L3 | SuperContig Scaffold6843: 20,552-25,742 | 684 | Ⅱ | na | na |
| Aet-M8D1R1 | SuperContig Scaffold6779: 40,262-42,421 | 719 | Ⅹ | na | na |
| Aet-M8D5M0 | SuperContig Scaffold3915: 328,257-329,687 | 476 | Ⅱ | na | na |
| Aet-N1QPS5 | SuperContig Scaffold242680: 3,509-7,511 | 792 | Ⅴ | na | na |
| Aet-N1QQB9 | SuperContig Scaffold116834: 14,814-22,199 | 763 | Ⅰ | na | na |
| Aet-N1QTL0 | SuperContig Scaffold268015: 2,943-5,030 | 695 | Ⅱ | na | na |
| Aet-N1QVU2 | SuperContig Scaffold43656: 9,089-14,833 | 607 | Ⅵ | na | na |
| Aet-N1QXD0 | SuperContig Scaffold42140: 5,426-7,441 | 671 | Ⅺ | na | na |
| Aet-N1QYC5 | SuperContig Scaffold47039: 48,759-51,392 | 703 | Ⅰ | na | na |
| Aet-N1QYP8 | SuperContig Scaffold35928: 88,552-90,685 | 668 | Ⅱ | na | na |
| Aet-N1QYQ3 | SuperContig Scaffold35928: 143,336-146,557 | 699 | Ⅱ | na | na |
| Aet-N1QZH7 | SuperContig Scaffold35528: 168,921-171,213 | 651 | Ⅱ | na | na |
| Aet-N1QZQ5 | SuperContig Scaffold36325: 62,160-67,676 | 690 | Ⅰ | na | na |
| Aet-N1R0I3 | SuperContig Scaffold35928: 110,528-112,709 | 615 | Ⅱ | na | na |
| Aet-N1R3W1 | SuperContig Scaffold35928: 97,329-100,113 | 627 | Ⅱ | na | na |
| Aet-R7W250 | SuperContig Scaffold168723: 29,141-31,379 | 713 | Ⅲ | na | na |
| Aet-R7WAW0 | SuperContig Scaffold30333: 25,310-27,313 | 667 | Ⅺ | na | na |
| Aet-R7WEK8 | SuperContig Scaffold34121: 6,037-8,055 | 672 | Ⅹ | na | na |
| Aet-R7WF04 | SuperContig Scaffold30333: 21,187-23,193 | 668 | Ⅹ | na | na |
| Aet-R7WFF4 | SuperContig Scaffold31432: 37,179-39,278 | 614 | Ⅲ | na | na |
| Aet-R7WGD4 | SuperContig Scaffold28814: 33,119-35,864 | 522 | Ⅳ | na | na |

Note: na means information not available.
